# Large Magnetoresistance in a Si-Based Double-Tunnel Junction with Purely Organic Radical Molecules

**DOI:** 10.1021/acs.nanolett.6c01526

**Published:** 2026-06-19

**Authors:** Jayanta Bera, Tuhin Shuvra Basu, Jannic Wolf, Haitao Zhang, Kazuhiro Marumoto, Yutaka Wakayama, Carmen Herrmann, Thomas Huhn, Ryoma Hayakawa

**Affiliations:** † Semiconductor Functional Device Group, Research Center for Materials Nanoarchitectonics (MANA), 52747 National Institute for Materials Science (NIMS), 1-1 Namiki, Tsukuba, Ibaraki 305-0044, Japan; ‡ Department of Chemistry, 26567 University of Konstanz, 78457 Konstanz, Germany; § Department of Materials Science, Institute of Pure and Applied Sciences, 13121 University of Tsukuba, Tsukuba, Ibaraki 305-8573, Japan; ∥ Institute for Inorganic and Applied Chemistry, 14915 University of Hamburg, Martin-Luther-King-Platz 6, 20146 Hamburg, Germany

**Keywords:** organic radicals, magnetoresistance, unpaired electrons, resonant tunneling, molecular orbitals, Si-based double-tunnel junctions

## Abstract

Organic radicals have shown promise for tunable and low-cost spintronic devices. However, integrating the radicals with a Si metal–oxide–semiconductor (MOS) structure remains a challenge. Here, we incorporate stable (4-(((2,5-bis­(2-(phenyl)­ethynyl)­phenyl)­carbonyl)­(methyl)­amino)-2,2,6,6-tetramethylpiperidin-1-yl)­oxidanyl (TEMPO-OPE) radicals in a Si-MOS-based double-tunnel junction and demonstrate a huge positive magnetoresistance of up to 400% at a magnetic field of 7 T and a temperature of 3 K. This goes along with a significant reduction of the differential conductance peak corresponding to the highest occupied molecular orbital (HOMO) of TEMPO-OPE under external magnetic fields. First-principles calculations suggest that the singly occupied molecular orbital can mix with the HOMO of TEMPO-OPE. This could lead to suppression of the HOMO conductance peak under magnetic fields and, thus, provide a possible origin of the large magnetoresistance. These findings suggest a path toward incorporating magnetic molecular functionalities into conventional Si devices, leading to large-scale integration of molecular spintronic devices.

## Graphical abstract

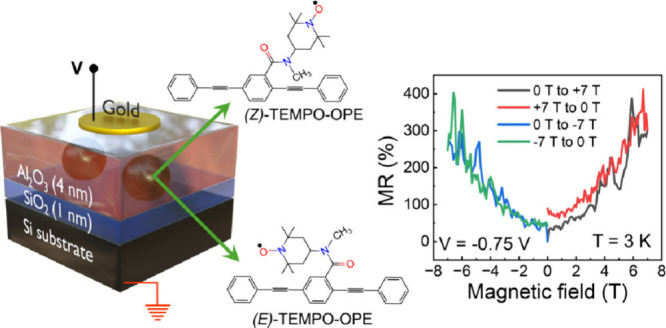

Molecular spintronics, which utilizes both the degree of freedom of spin and charge, is an emerging field based on spin-dependent carrier transport through individual molecules or their assemblies.^1^ This feature is expected to offer significant applications, including low-power memory, spin-based logic, and quantum computing.^2–10^ Stable organic radicals possess a paramagnetic nature owing to their open-shell system with unpaired electrons. Due to their light-element compositions with carbon, hydrogen, nitrogen, and oxygen, they exhibit low spin–orbit coupling and weak hyperfine interactions.^11^ Therefore, organic radicals have a longer spin coherence time (∼7 μs) compared to inorganic counterparts, like transition metal complexes and single-molecule magnets, even at room temperature.^12–14^ This feature contributes to the protection of the information stored in the electronic spin.^15,16^ These attractive features of purely organic radicals make them promising candidates for molecular spintronic devices.

The spin-dependent carrier transport through individual molecules and their assemblies was generally investigated using break-junction techniques, conductive probe atomic force microscopy, and scanning tunneling microscopy (STM) techniques.^17–32^ For example, Liu et al. observed Kondo resonance in 1,3,5-triphenyl-6-oxoverdazyl (TOV) molecules using scanning tunneling spectroscopy (STS), which arises due to the interaction between a localized spin of TOV and conduction electrons of metal electrodes.^17^ Müllegger et al. demonstrated Kondo resonance in α,γ-bisdiphenylene-β-phenylallyl (BDPA) molecules on Au(111) surfaces.^18^ Frisenda et al. detected the presence of an unpaired electron spin of a polychlorotriphenylmethyl (PTM) radical using mechanically controlled break junction (MCBJ) and electromigrated break junction techniques.^24^ Additionally, large magnetoresistance (MR) was observed in molecular junctions with non-magnetic molecules, such as fullerene (C_60_) and benzene, using magnetic electrodes.^33,34^ Similar MR effects were visualized in transition metal complexes in single-molecular junctions formed by STM.^22,35^ Furthermore, Mitra et al. observed Kondo resonance and MR in the molecular junctions with PTM radicals^25^ or Blatter radicals^26^ using MCBJ techniques. However, the above-mentioned techniques are not adopted for large-scale integration of molecular spintronics devices. To overcome this problem, we demonstrated Si-based double-tunnel junctions, where isolated molecules are sandwiched as quantum dots (QDs) between two oxide layers of a metal–oxide–semiconductor (MOS) structure.^36–38^ In our previous work, we demonstrated resonant tunneling via the singly occupied molecular orbital (SOMO) of adamantyl nitronyl nitroxide *p*-terphenyl (NN-TP) radicals in the Si-based double-tunnel junction.^39^ This result clarified the survival of unpaired electrons of the organic radicals in the device structure. However, we had not observed obvious magnetoresistance (MR) in the samples with NN-TP.

In this work, we investigated magnetic-field-dependent carrier transport via TEMPO-OPE radicals incorporated into the Si-based double-tunnel junction. TEMPO-OPE possesses an unpaired electron on the TEMPO radical, which is not conjugated with the π orbitals of the OPE backbone molecule (Figure 1a). Rather, the radical part of TEMPO-OPE is electrically isolated from the main transport channel, in contrast to the case of NN-TP. This property is expected to preserve the localization of the unpaired electron on the TEMPO radical and to enable the weak coupling to the π orbitals of the OPE backbone due to the spatial proximity between the TEMPO radical and the OPE backbone. Actually, we have achieved large MR values of up to 287% at a magnetic field of 4 T in the single-molecule junction formed by a MCBJ technique in our previous study.^27^ This finding inspired us to explore the carrier transport via TEMO-OPE in the Si-based double-tunnel junction under magnetic fields. Although similar examples, e.g., grafting of magnetic molecules onto Si substrates, have been reported, no MR effects have appeared in Si-based molecular junctions.^40–42^ Here, we observed a huge positive MR of up to 400% in the TEMPO-OPE sample under an applied magnetic field of 7 T and a temperature of 3 K. This can be attributed to a significant reduction in the differential conductance (d*I*/d*V*) peak corresponding to the highest occupied molecular orbital (HOMO) of TEMPO-OPE under magnetic fields. In contrast, no significant MR was observed in non-radical OPE (closed-shell type) samples. Our findings thus have the potential to integrate magnetic functions of organic radicals into large-scale-integrated Si devices in the future.

**Figure 1:**
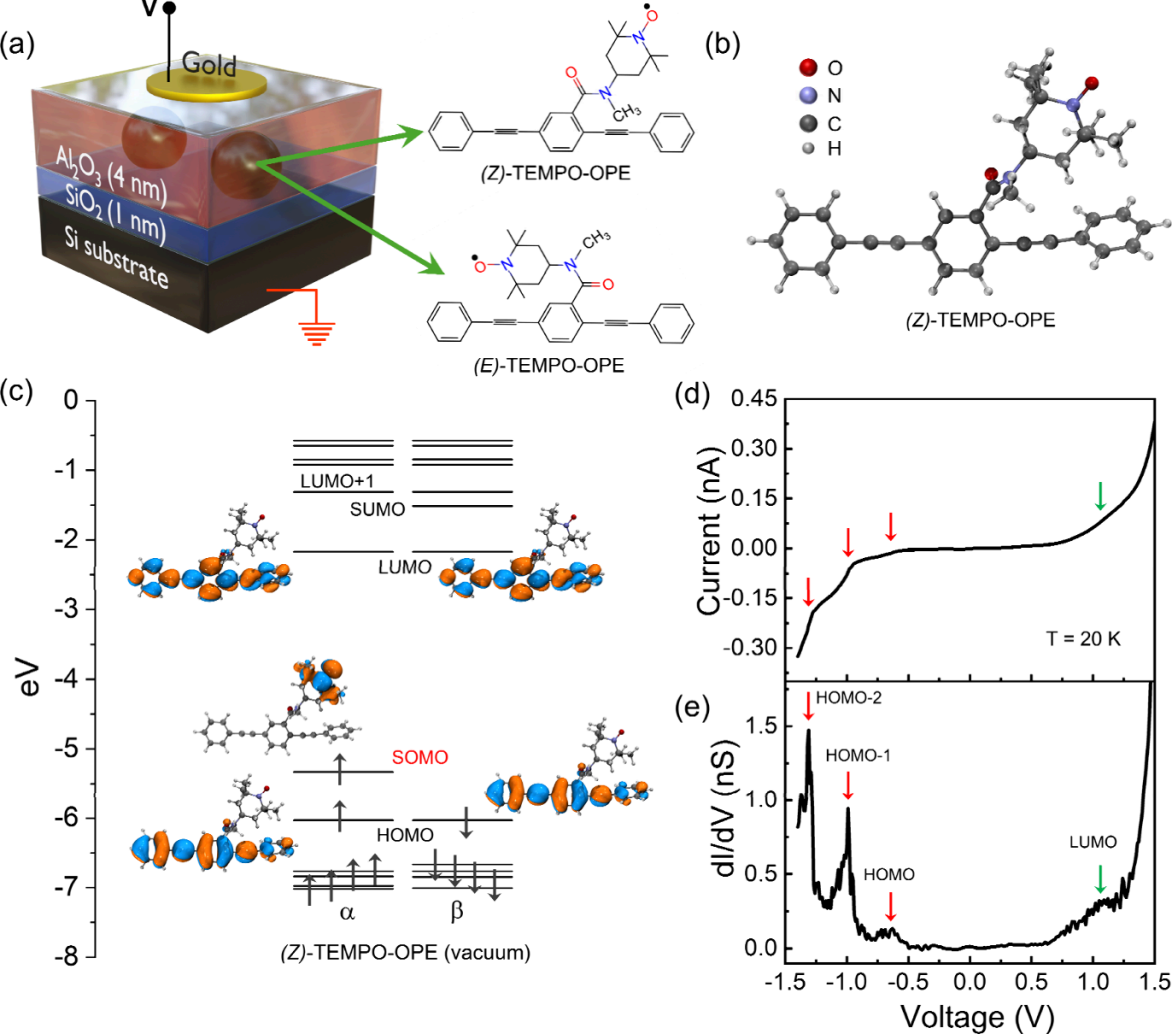
(a) Schematic illustration of the Si-based double-tunnel junction with TEMPO-OPE molecules. (b) Optimized structure of (*Z*)-TEMPO-OPE in vacuum. (c) Molecular orbital energy diagram and corresponding isosurfaces for (*Z*)-TEMPO-OPE in vacuum. (d) *I*–*V* characteristic and (e) corresponding d*I*/d*V* curve of the Si-based double-tunnel junction with TEMPO-OPE molecules measured at a temperature of 20 K.

A Si-based double-tunnel junction with TEMPO-OPE molecules embedded as quantum dots was formed on a highly doped Si (p+) substrate (Figure 1a). Here, individual TEMPO-OPE molecules were embedded in an Al_2_O_3_ layer, in which the number of molecules was estimated to be in the order of 10^12^–10^13^ cm^–2^ based on our previous study.^36^ The insulating layers (SiO_2_ and Al_2_O_3_) function as the tunnel barriers, while the p+ Si substrate serves as the bottom electrode. The detailed formation processes are shown in section 1.2 of the Supporting Information. It is noteworthy that the TEMPO-OPE molecule exhibits two isomeric configurations, namely, (*Z*)- and (*E*)-TEMPO-OPE, depending on the spatial configuration of the TEMPO group with respect to rotation around the amide bond (C–N) that links the TEMPO substituent to the backbone. Therefore, we consider both isomeric configurations in the Si-based double-tunnel junction when modeling their properties by first-principles methods.

Figure 1b and c shows the optimized molecular structure and energy diagram of an isolated (*Z*)-TEMPO-OPE molecule evaluated by Kohn–Sham density functional theory (KS-DFT).^43^ We focus here on (*Z*)-TEMPO-OPE as it tends to be more energetically preferable than (*E*)-TEMPO-OPE (Table S3) when adsorbed on an oxide surface due to a larger contact area (it should be kept in mind that, in their isolated form, the two isomers are very similar in terms of their total energy, molecular orbital energies, and vibrational modes (sections 5 and 6 of the Supporting Information) and that the isomers’ exact atomic configuration when embedded in an oxide is unknown; thus, both could be present in the experiment). The optimized molecular structure, the molecular orbital energy diagram of (*E*)-TEMPO-OPE, and the detailed computational methods are described in section 5 of the Supporting Information. The SOMO is mainly located on the TEMPO radical part in both the isomeric configurations, corresponding to a π*_N–O_ orbital.

Figure 1d and e depicts a typical current (*I*)–voltage (*V*) characteristic and the corresponding d*I*/d*V* curve of the TEMPO-OPE sample measured at a temperature of 20 K, where the p+ Si substrate was grounded and a voltage was applied to the top gold electrode. Three distinct “staircase”-like signals were observed at the negative voltage range, and one “staircase” was visible in the positive voltage range in the *I*–*V* curve (Figure 1d). The corresponding d*I*/d*V* peaks were observed at −0.63, −0.99, −1.31, and 1.12 V in Figure 1e. Similar d*I*/d*V* peaks were observed in 25 out of a total 119 devices, corresponding to a yield of approximately 21%, which is consistent with that in the devices with NN-TP molecules.^39^ Conversely, no d*I*/d*V* peaks appeared in the reference samples without any molecules (Figure S11b). Thus, the prominent d*I*/d*V* peaks in Figure 1e indicate that the TEMPO-OPE molecules remain intact in the double-tunnel junction.

Our previous studies revealed that the tunneling current reflecting the occupied MOs arises in the negative voltage range due to the resonant tunneling of holes from the underlying Si substrate to the embedded molecules.^36,38,39^ Likewise, unoccupied MOs arise in the positive voltage range owing to resonant tunneling of electrons from the underlying Si substrate to the embedded molecules. Given the peak assignment in the non-radical OPE device (Figures S9b and S10d), the d*I*/d*V* peaks visible at −0.63, −0.99, and −1.31 V, which are indicated by red arrows in Figure 1d and e, can be ascribed to the HOMO, HOMO–1, and HOMO–2 of TEMPO-OPE molecules, respectively. The d*I*/d*V* peak at 1.12 V, which is indicated by a green arrow, corresponds accordingly to the lowest unoccupied molecular orbital (LUMO) of TEMPO-OPE molecules. The mean values of peak positions were estimated to be −0.68 ± 0.12 V for the HOMO and 1.08 ± 0.09 V for the LUMO, respectively, from the statistical d*I*/d*V* measurements in the TEMPO-OPE devices (Figure S10c). Accordingly, the HOMO–LUMO gap was estimated at 1.8 eV, which is comparable to that of non-radical OPE (1.4 eV) (Figure S10d).

The carrier transport of Si-based double tunnel junctions with TEMPO-OPE molecules was examined under magnetic fields to clarify the role of the unpaired electron spin. Figure 2a shows the *I*–*V* characteristics of the sample under magnetic fields ranging from 0 to 7 T, where magnetic fields were applied in the perpendicular direction to the sample plane. The measurement temperature was fixed at 3 K. It is noted that 10-point-data smoothing with the Savitzky–Golay method was implemented in Figure 2a and b to eliminate large noise. The comparison of raw data and the smoothed curves is given in Figure S12. Significantly, the tunneling current via the HOMO level was strongly reduced by magnetic fields, as indicated by the red arrow in Figure 2a. The current level was returned to the original one by the reduction of the magnetic field from 7 to 0 T (Figure S13). In contrast, no changes in the tunneling current through the LUMO level were observed under the same magnetic fields.

**Figure 2:**
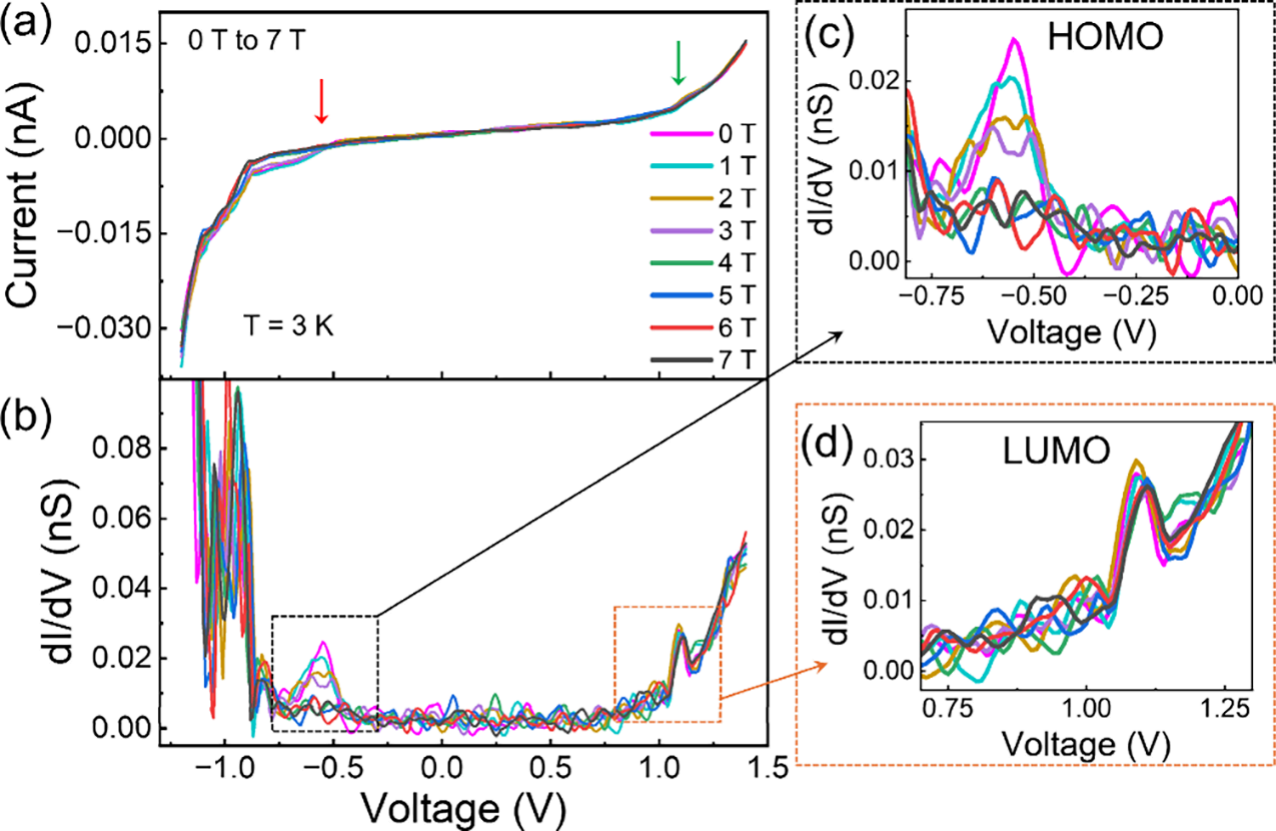
(a) *I*–*V* characteristics and (b) corresponding d*I*/d*V* curves of TEMPO-OPE samples under magnetic fields ranging from 0 to 7 T. The d*I*/d*V* peaks corresponding to the HOMO and LUMO levels of TEMPO-OPE molecules are shown in enlarged views with (c) black and (d) orange dotted rectangles, respectively. The magnetic field was applied perpendicular to the sample plane.

This intriguing occurrence was more clearly visible in the d*I*/d*V* curves. As shown in Figure 2b, the d*I*/d*V* peak corresponding to the HOMO level was closely suppressed under magnetic fields (Figure 2c). Consequently, the d*I*/d*V* peak disappeared in magnetic fields of above 3 T. This reduction in the HOMO d*I*/d*V* peak is also confirmed in two-dimensional (2D) color maps of d*I*/d*V* curves as a function of magnetic fields (Figure S14). On the other hand, no significant changes were observed in the d*I*/d*V* peak attributed to the LUMO level (Figure 2d). Moreover, the same behavior, namely, the reduction in the HOMO d*I*/d*V* peak, was observed in a different device (Figure S15). Such variations in tunneling currents under magnetic fields did not appear in both samples with non-radical OPE molecules (Figure S9c and d) and without any molecules (Figure S11a and b).

In order to probe the exact variations in tunneling current via the HOMO and LUMO levels, we performed magnetoresistance (MR) measurements on our samples at fixed voltages. MR values were calculated as $\left(\frac{R_{M}-R_{0}}{R_{0}}\right)\times 100\left(\%\right)$ , where *R*_M_ and *R*_0_ are the resistances obtained at ±7 and 0 T, respectively. The MR was measured with the following sequence of magnetic fields: 0 → 7 → 0 → −7 → 0 T. Figure 3a and b shows the variation in MR curves obtained at −0.75 and 1.2 V, respectively, as a function of applied magnetic fields, where the measurement temperature was fixed at 3 K. A large positive MR value of up to 400% was observed in TEMPO-OPE samples at −0.75 V, which corresponds to the HOMO level of the TEMPO-OPE molecules. The observed MR value in the TEMPO-OPE sample is higher than the previously reported experimental values obtained using organic radicals and metal phthalocyanines in single-molecule junctions.^22,23,25–27^ In contrast, no significant MR was observed at 1.2 V, which agrees with the LUMO level of the TEMPO-OPE molecules. For comparison, we carried out MR measurements on the non-radical OPE samples (Figure S9e and f) as well as the sample without molecules (Figure S11c and d). In both cases, we did not observe any MR effect. These findings clarify that the MR effect in the TEMPO-OPE sample is caused by the TEMPO radical group.

**Figure 3:**
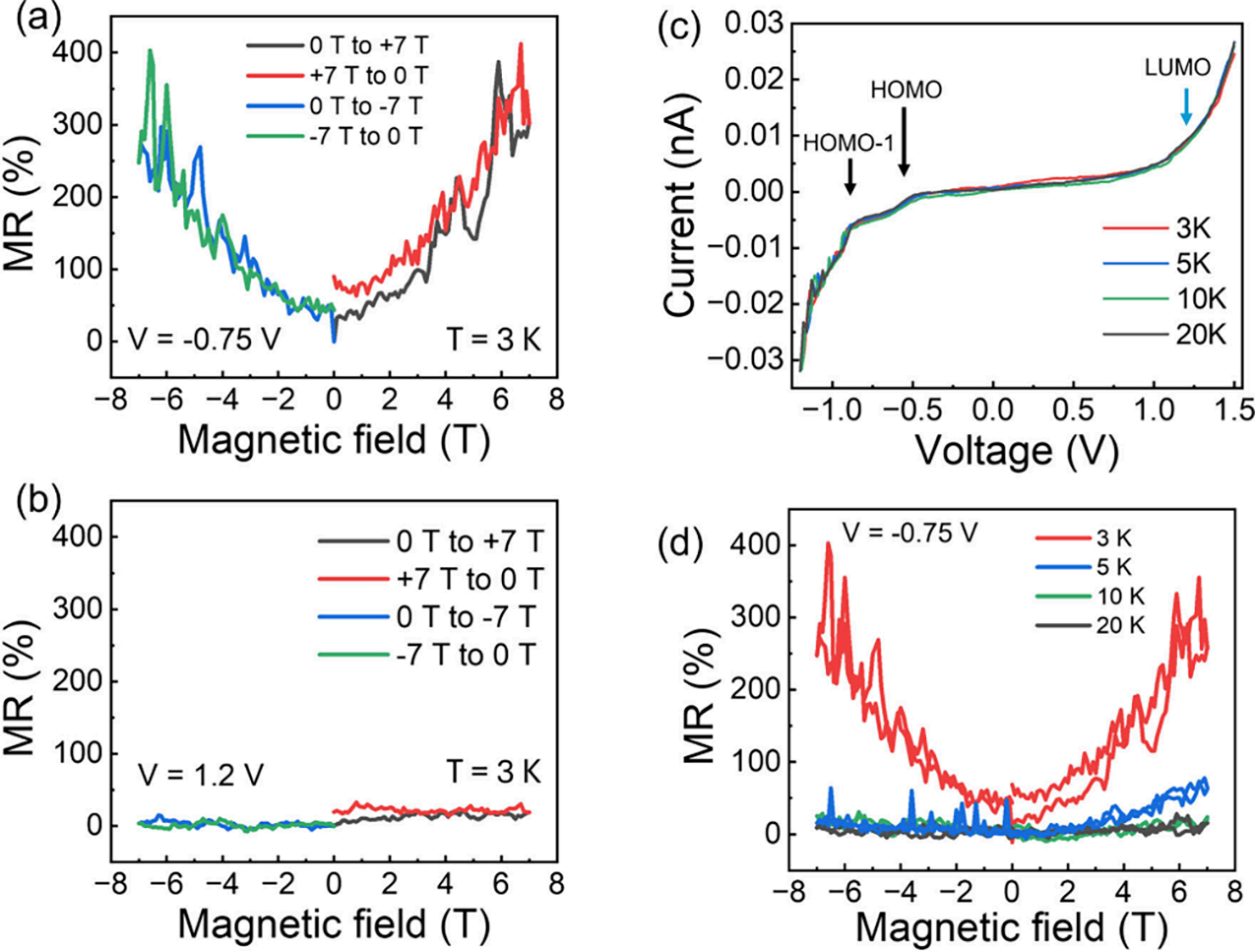
Variation of MR in TEMPO-OPE samples with an applied magnetic field at (a) −0.75 V, which corresponds to the HOMO peak, and (b) 1.2 V, which agrees with the LUMO peak. The magnetic field varied within the range of ±7 T. The magnetic field sweep sequence was as follows: 0 to +7 T (black line) → +7 to 0 T (red line) → 0 to −7 T (blue line) → −7 to 0 T (green line). The measurement temperature was fixed at 3 K in panels a and b. (c) *I*–*V* and (d) MR curves obtained at −0.75 V (HOMO) of TEMPO-OPE samples measured at a varied temperature ranging from 3 to 20 K.

Figure 3c and d depicts the *I*–*V* and MR curves of TEMPO-OPE samples measured at a varied temperature ranging from 3 to 20 K. No significant changes in *I*–*V* curves were observed with the temperature changed. However, a drastic reduction in the MR value was observed when the temperature increased to 5 K, eventually disappearing at temperatures higher than 5 K.

Next, we discuss the possible origins of the large positive magnetoresistance (MR) in the double-tunnel junction with TEMPO-OPE molecules. Such a large MR of up to 400% in the double-tunnel junction with radical molecules has not yet been reported. A negative MR was observed by Sugawara et al. in Au nanoparticles connected via a nitronyl nitroxide radical molecule. The negative MR was attributed to a decrease in spin-flip scattering because an applied magnetic field aligns localized spins, thereby limiting the spin-flip scattering of conduction electrons with an increasing magnetic field.^44,45^ In our case, we observed only positive MR, and thus, this scenario is excluded. Mitra et al. reported a positive MR of 140% at a magnetic field of 6 T in single-molecule junction of perchlorotriphenylmethyl radicals, where asymmetrically coupled junctions exhibited Kondo resonance, while symmetrically coupled junctions showed high MR with both positive and negative signs. The origin of MR was attributed to the spin polarization of the SOMO in combination with spin-dependent scattering at metal–molecule interfaces.^25^ In TEMPO-OPE molecules, the position of the radical component is far from the OPE backbone, resulting in equal transmission probabilities for both up and down spins for transport pathways through the backbone.^27^ Therefore, spin polarization is less likely in TEMPO-OPE molecules. Previous experiments suggested that a large MR value of up to 278% at a magnetic field of 4 T in single-molecule junctions of TEMPO-OPE could originate from a reduced coupling between the molecular orbitals and the metal electrodes.^27^ However, in this present study, the TEMPO-OPE molecules are not directly coupled to the metal electrodes, and therefore, this scenario is also ruled out. Warner et al. reported MR (both positive and negative) in an asymmetrically coupled single-molecular junction, where an iron phthalocyanine (FePc) molecule on a thin insulator layer (copper nitride) on a Cu(001) surface was probed by a STM tip (PtIr). The system configuration (metal/vacuum/molecule/insulator/metal) is similar to our Si-based double-tunnel junction embedding TEMPO-OPE. The MR was assumed to arise from the shift of negative differential resistance (NDR) in d*I*/d*V* spectra under a varied magnetic field. The shift of the NDR peak was attributed to the spin-polarized and non-degenerate resonant levels caused by the exchange splitting between the up and down spins of Fe d states.^22^ However, we did not observe any such shift in the d*I*/d*V* peaks; rather, the HOMO peak was suppressed and eventually disappeared under the magnetic field. The magnetic-field-dependent carrier transport in our sample is rather attributed to the fading away of the d*I*/d*V* peak corresponding to the HOMO level.

To validate our results, we have performed DFT-based calculations of the effective Kohn–Sham single-particle energy levels (MOs) and density of states (DOS) of the TEMPO-OPE molecule embedded in a double-layer system composed of SiO_2_ and Al_2_O_3_. The simulations for the molecule in the tunnel junction were performed employing the PBE functional. The calculation details are described in section 7 of the Supporting Information. Figure 4a and Figure S7a show the optimized structure of (*Z*)- and (*E*)-TEMPO-OPE, respectively, where the molecules are sandwiched between hydroxyl (OH^–^)-terminated SiO_2_ and Al_2_O_3_ layers. Termination of SiO_2_ and Al_2_O_3_ surfaces by the OH^–^ group is confirmed from X-ray photoelectron spectroscopy measurements (Figures S3 and S4). The spin-density isosurfaces of (*Z*)- and (*E*)-TEMPO-OPE embedded between the hydro-α-SiO_2_ and the hydro-α-Al_2_O_3_ layers are shown in Figure 4b and Figure S7b. The spin density is concentrated on the nitroxyl part of the TEMPO group, which means that the open-shell nature of TEMPO-OPE is well-preserved, even in insulating layers. The total DOS of the hydro-α-Al_2_O_3_/(*Z*)-TEMPO-OPE/hydro-α-SiO_2_ structure and partial DOS (PDOS) of (*Z*)-TEMPO-OPE in the sandwiched structure are shown in Figure 4c and d, respectively. The DOS and PDOS curves indicate that energy levels of (*Z*)-TEMPO-OPE molecules are located in the energy gaps of the two insulating layers and that they are well-separated from the continuum DOS of SiO_2_ and Al_2_O_3_. The estimated HOMO–LUMO gap obtained from the PDOS curve (Figure 4d) is approximately 2.5 eV, which slightly differs from the value of 1.8 eV, as estimated from the experimental d*I*/d*V* curve (Figure S10c). This difference would be caused by the assumption of an idealized junction configuration. In this calculation, a single TEMPO-OPE is located at the interface of the two oxide layers. However, in the real device, individual molecules are completely surrounded by the Al_2_O_3_ layer, and further, each molecule can interact with the other molecules. The detailed discussion is provided in section 9 of the Supporting Information.

**Figure 4:**
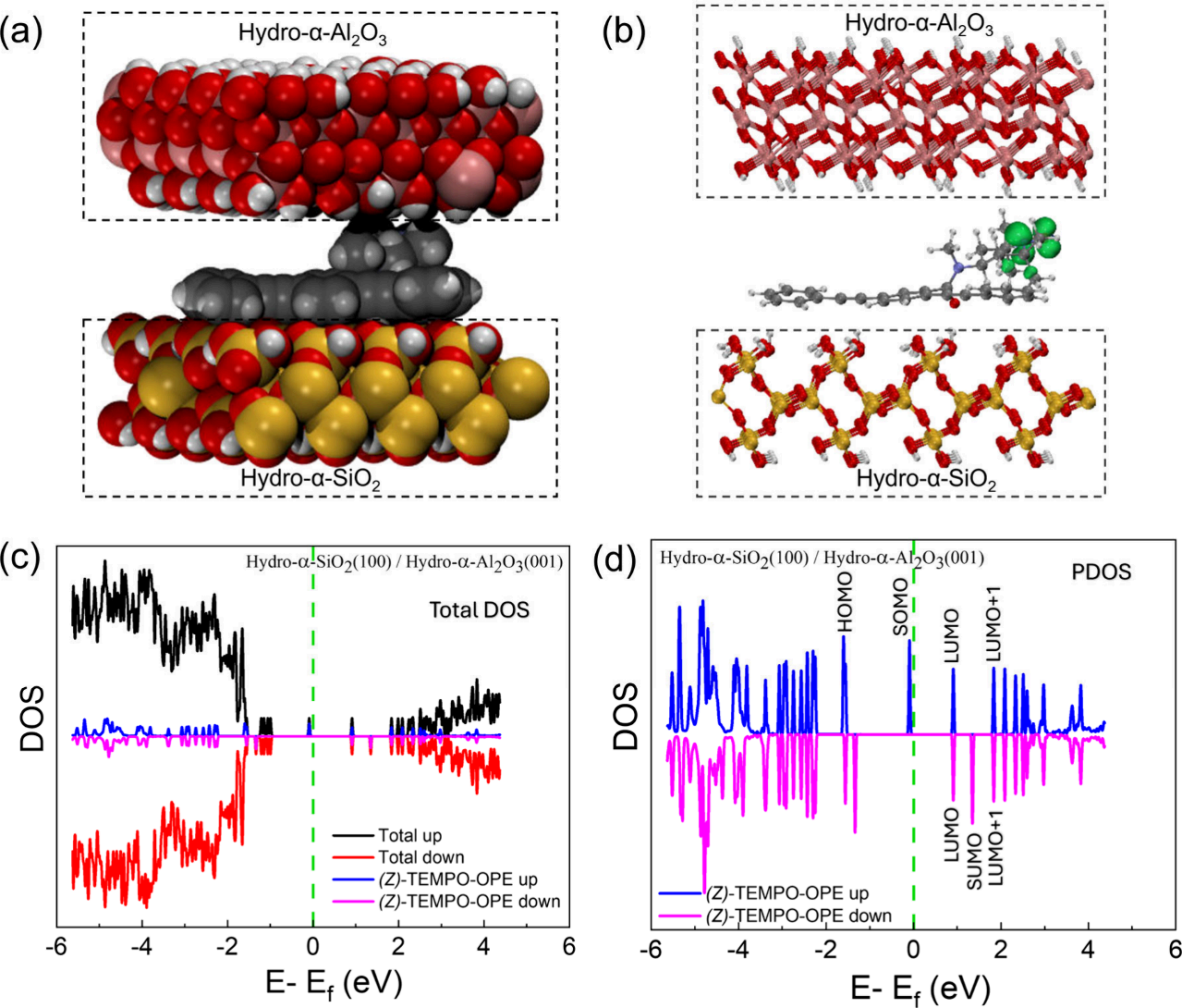
(a) Optimized structure, (b) spin-density isosurfaces, (c) total DOS, and (d) partial DOS of (*Z*)-TEMPO-OPE embedded between the hydro-α-SiO_2_ and hydro-α-Al_2_O_3_ layers. The Fermi level is set to 0 eV, as shown by the green dashed line in DOS plots. The states below the Fermi level are labeled as the occupied molecular orbital, and the states above Fermi level are labeled as the unoccupied molecular orbital of TEMPO-OPE.

The most important observation from the PDOS of TEMPO-OPE is that the SOMO is energetically split into an occupied spin-up part and an unoccupied spin-down part, with the occupied spin-up part located close to the Fermi level of the junction (Figure 4d and Figure S7d). It is noted that related aspects of close spatial proximity, with a perpendicular molecular configuration and potentially different mechanistic implications, were discussed in our previous work on TEMPO-OPE in gold break junctions.^27,46^ Although we likely did not observe the SOMO level in the experimental d*I*/d*V* spectra in our TEMPO-OPE samples due to its localization on TEMPO’s NO part, the SOMO may interact with the HOMO of TEMPO-OPE, e.g., through the following mechanisms.

A first possible scenario is derived from the Zeeman effect. When a magnetic field is applied, the SOMO energy is shifted by the Zeeman effect. This change in the SOMO would be translated to the HOMO but not to the LUMO. In the DFT calculations of TEMPO-OPE in the tunnel junction, the HOMO is extended onto the radical part in (*Z*)-TEMPO-OPE [and to a lesser extent in (*E*)-TEMPO-OPE] and could thus interact with the localized SOMO (Tables S4 and S6). Conversely, such mixing is much less pronounced for LUMO. Given the apparent sensitivity of the mixing between backbone-centered and radical-centered contributions in the HOMO, it may be that magnetic-field-induced shifts in the SOMO can also change the shapes of the orbitals, in particular the HOMO. This could then lead to the observed suppression of the HOMO d*I*/d*V* peak with an increased magnetic field.

Another scenario is the conformation or orientation change of TEMPO-OPE molecules under magnetic fields. Although the molecules are embedded in the oxide layers, a substantial portion of the molecules could be able to rearrange this way. Indeed, we confirmed a similar conformation change with photochromic molecules even in the oxide layers in our previous work,^37^ and magnetic-field-induced structural changes have also been reported in the literature.^47–49^ Such changes in both the geometrical and electronic structures could be possible in a magnetic field, affecting the subtle coupling between the SOMO and HOMO.

Both mechanisms would be expected to exhibit a temperature-dependent transport. For the first scenario, a Zeeman splitting of a single unpaired electron would not be expected to be measurable at more than 10 K. For the second one, the conformational or orientational effect under magnetic fields would likely be overridden by thermal fluctuations. However, it should be noted that the suggested mechanisms are not definitive, and validating them would require further investigation.

In summary, we evaluated magnetic-field-dependent carrier transport via discrete molecular levels of TEMPO-OPE molecules incorporated into a Si-based double-tunnel junction. A significant reduction of the d*I*/d*V* peak corresponding to the HOMO level of TEMPO-OPE molecules was observed under external magnetic fields. A large positive MR of 400% at the maximum was achieved in TEMPO-OPE samples at a magnetic field of 7 T and a temperature of 3 K. However, no significant MR was observed in reference samples with non-radical OPE and without any molecules. DFT analysis suggests that magnetic-field-induced changes in the SOMO or in the conformation of the molecules can translate to the HOMO and the mixing between backbone-centered and radical-centered contributions in the HOMO. These effects may lead to the observed suppression of the HOMO conductance peak under a magnetic field. Thus, our approach provides an effective way to integrate magnetic functionality into Si devices, offering great potential for large-scale integration of molecular spintronic devices.

## Supplementary Material



## Notes

Experimental methods, FTIR spectra and ESR measurements of TEMPO-OPE films, XPS measurements of Si-based double-tunnel junctions, first-principles calculations for molecular orbitals of TEMPO-OPE in vacuum as well as in the double-tunnel junction, vibrational spectra and structural stabilities of optimized (*Z*)- and (*E*)-TEMPO-OPE, *I*–*V* and d*I*/d*V* curves of non-radical OPE samples without the TEMPO group and reference double-tunnel junction without any molecules under external magnetic fields, histograms of the tunneling current and d*I*/d*V* peak positions corresponding to the HOMO and LUMO in TEMPO-OPE and non-radical OPE samples, raw and 10-point smoothed data of *I*–*V* and d*I*/d*V* of TEMPO-OPE samples, and *I*–*V* and d*I*/d*V* of TEMPO-OPE samples under magnetic fields (PDF)
