# Supplementary material for: Large Magnetoresistance in a Si-Based Double-Tunnel Junction with Purely Organic Radical Molecules
Source: Nano Lett. 2026 Jun 19;26(25):8257–64. doi: 10.1021/acs.nanolett.6c01526 (PMC13329981; doi:10.1021/acs.nanolett.6c01526)
Supplement: Supplementary file 1 [file nl6c01526_si_001.pdf]

Supporting Information for

Large magnetoresistance in a Si-based double-tunnel junction with purely organic radical molecules

Jayanta Bera[†], *Tuhin Shuvra Basu*[†], *Jannic Wolf*[※], *Haitao Zhang*[§], *Kazuhiro Marumoto*[‡], *Yutaka Wakayama*[†], *Carmen Herrmann*[§]*, *Thomas Huhn*[※]*, and *Ryoma Hayakawa*[†]*

[†] Semiconductor Functional Device Group, Research Center for Materials Nanoarchitectonics (MANA), National Institute for Materials Science (NIMS), 1-1 Namiki, Tsukuba, Ibaraki, 305-0044, Japan

[※] Department of Chemistry, University of Konstanz, Konstanz, 78457, Germany

[‡] Department of Materials Science, Institute of Pure and Applied Sciences, University of Tsukuba, Tsukuba, Ibaraki, 305-8573, Japan

[§] Institute for Inorganic and Applied Chemistry, University of Hamburg, Martin-Luther-King-Platz 6, 20146 Hamburg, Germany

1. Experimental Methods

1.1. The synthesis process of TEMPO-OPE

A mixture of (4-(((2,5-diiodophenyl)carbonyl)(methyl)-amino)-2,2,6,6-tetramethylpiperidin-1-yl)oxidanyl[1] (405 mg, 0.748 mmol), ethynylbenzene (179 mg, 1.750 mmol) and triethylamine (5 ml) was degassed by three freeze pump cycles. $PdCl_2(PPh_3)_2$ (10 mg) and CuI (1.80 mg) were added and the solution was stirred at 50 °C for 10 h. Volatiles were removed *in vacuo* and the residue was partitioned between dichloromethane (DCM) and H_2O. After separation of the phases the aqueous phase was further extracted with dichloromethane. The combined DCM-phases were dried over $MgSO_4$ and the solvent was removed. The crude product was purified by column chromatography (25 g SiO_2, DCM) to yield the title compound as salmon-colored solid (224 mg, 61%).

^{13}C-NMR (100 MHz, acetone-d_6): δ = 168.54, 167.12, 139.06, 138.35, 134.02, 133.78, 133.30, 133.01, 132.56, 132.20, 131.73, 131.61, 131.32, 130.98, 130.83, 130.60, 130.32, 128.45, 128.26, 128.14, 128.03, 127.70, 127.58, 127.33, 127.20, 127.13, 124.22, 123.98, 123.52, 123.02, 122.85, 122.03, 121.63, 116.41, 116.04, 93.74, 93.23, 90.05, 89.88, 88.23, 88.07, 87.47, 87.31 ppm, (signals of the oxidanyl-part not detectable, mixture of *(E)*- and *(Z)*-isomers); Anal. Calcd for $C_{33}H_{33}N_2O_2^{\cdot}$: 80.95% (C), 6.79% (H), 5.72% (N), Found: 80.67% (C), 5.59% (H), 5.84% (N).

1.2. *Device Fabrication*

Si-based double-tunnel junctions with TEMPO-OPE molecules were formed according to our previous work.[2,3] First, circular holes with a diameter of 100 μm were formed on a highly doped p-type Si substrate (< 0.02 Ωcm) with a 300-nm-thick SiO_2 layer by laser lithography and reactive ion etching systems. The hole patterns reached the bare Si surface. Then, an ultrathin SiO_2 (1 nm) film, which serves as a bottom tunneling barrier, were grown on the substrate in a furnace by annealing at 773 K for 2 minutes in an oxygen atmosphere. Subsequently, TEMPO-OPE molecules were thermally deposited on the SiO_2/Si substrate in a thermal evaporation

system at high vacuum condition (5×10^{-7} Pa). Here, we confirmed that TEMPO-OPE molecules were not decomposed in the thermal evaporation process using Fourier transform infrared spectroscopy (**Figure S1**) and electron spin resonance (**Figure S2**) measurements. Next, the molecules were covered by a 4-nm-thick Al_2O_3 layer using an atomic layer deposition (ALD) process without breaking the high vacuum condition. The Al_2O_3 layer serves as an upper tunneling barrier and also protects the molecules from exposure to air. Finally gold circular electrodes with a diameter of 500 μm were deposited using a thermal deposition system.

1.3. *Electrical and Magnetic Measurements*

The fundamental $I-V$ and dI/dV curves of the devices with TEMPO-OPE molecules were measured using a semiconductor device analyzer (Keysight Technology B2912B) and a six-probe system (Nagase Techno-Engineering Co. Ltd.) in the condition without magnetic fields. In these measurements, the voltages were applied in the top gold electrodes, while the p+ Si substrates were grounded. Most measurements were conducted at 20 K. Then, the samples were transferred to a variable temperature cryostat system (Oxford Instruments) to evaluate magnetic-field-dependent carrier transport of TEMPO-OPE molecules. The magnetic field was applied up to 7 T with a super conducting magnet. The measurement temperature varied in the range of 3 to 20 K.

2. FT-IR spectra of TEMPO-OPE thin film on SiO_2/Si substrate

Figure S1 shows a typical FT-IR spectrum of a TEMPO-OPE film formed on a 1-nm-thick SiO_2/Si substrate. The spectrum indicates that the TEMPO group was not decomposed upon the deposition of the molecules on SiO_2 layer.

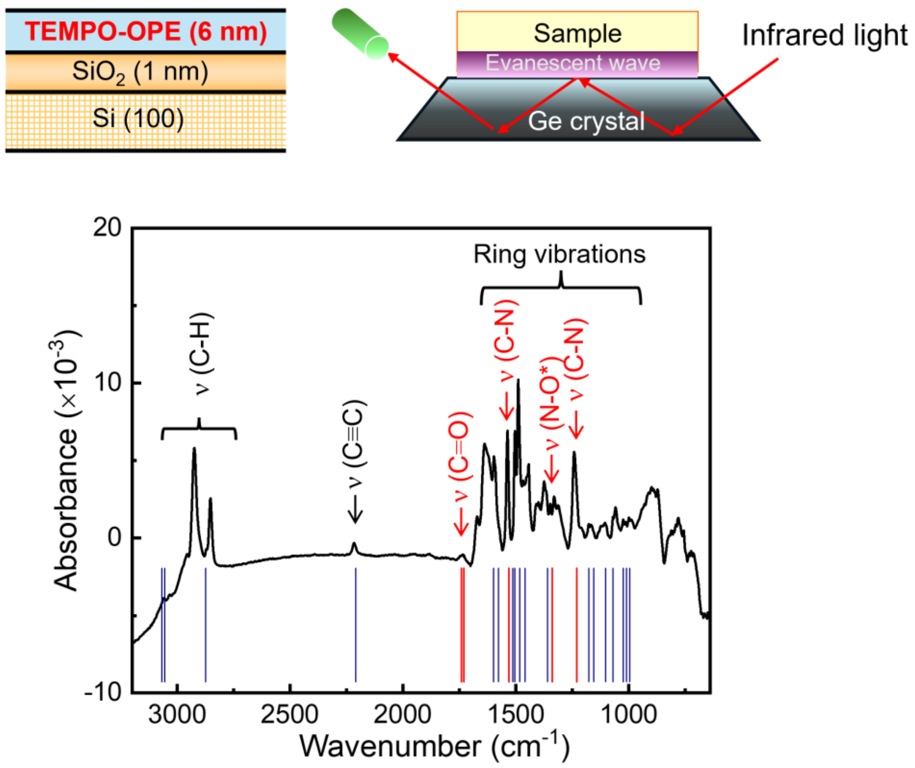

Figure S1. FT-IR spectrum of a TEMPO-OPE thin film grown on a SiO_2/Si substrate.

3. Electron Spin Resonance (ESR) measurements of TEMPO-OPE molecules

Figure S2a and **S2**b show electron spin resonance (ESR) spectra of a TEMPO-OPE solution and a TEMPO-OPE thin film deposited on a quartz substrate using a thermal evaporation technique. In both cases, the almost same g-factor values were observed, indicating that the TEMPO-OPE molecules were not decomposed during the thermal evaporation process.

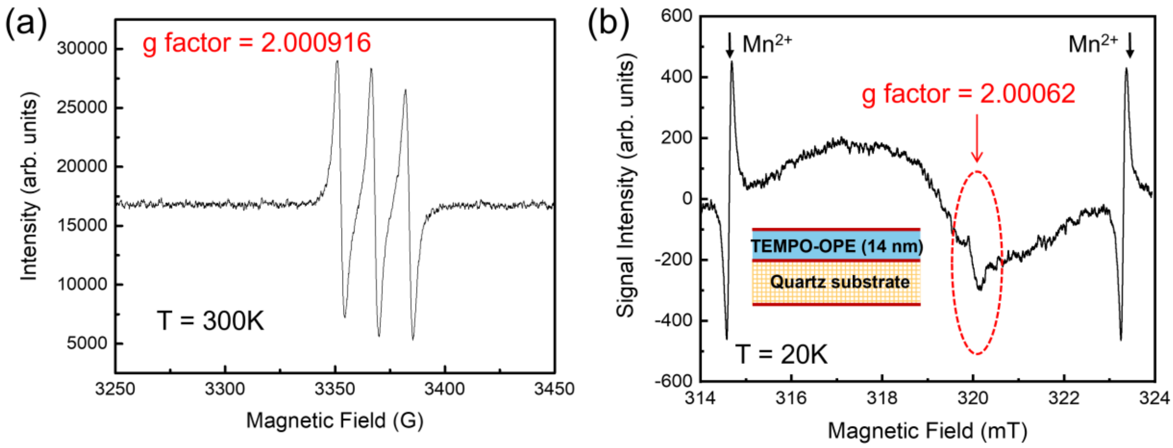

Figure S2. ESR spectra of (a) TEMPO-OPE solution and (b) TEMPO-OPE thin film grown on quartz substrate.

4. X-ray Photoelectron Spectroscopy (XPS) measurements of Si-based double-tunnel junctions

During the ALD process water vapor was introduced. Therefore, the hydroxyl (OH^-) group is expected to be present on the surfaces of SiO_2 and Al_2O_3 layers consisting of double-tunnel junctions. To confirm the presence of OH^- group, we performed XPS measurement in a double-tunnel junction ($Al_2O_3/SiO_2/Si$) (**Figure S3**a–e). The XPS spectra exhibited evidence of OH^- group (**Figure S3**d) in the $Al_2O_3/SiO_2/Si$ sample. In contrast no OH^- group was observed only in SiO_2/Si sample (**Figure S4**). These results indicate that Al_2O_3 and SiO_2 surface are terminated by hydroxyl group in our Si-based double-tunnel junction.

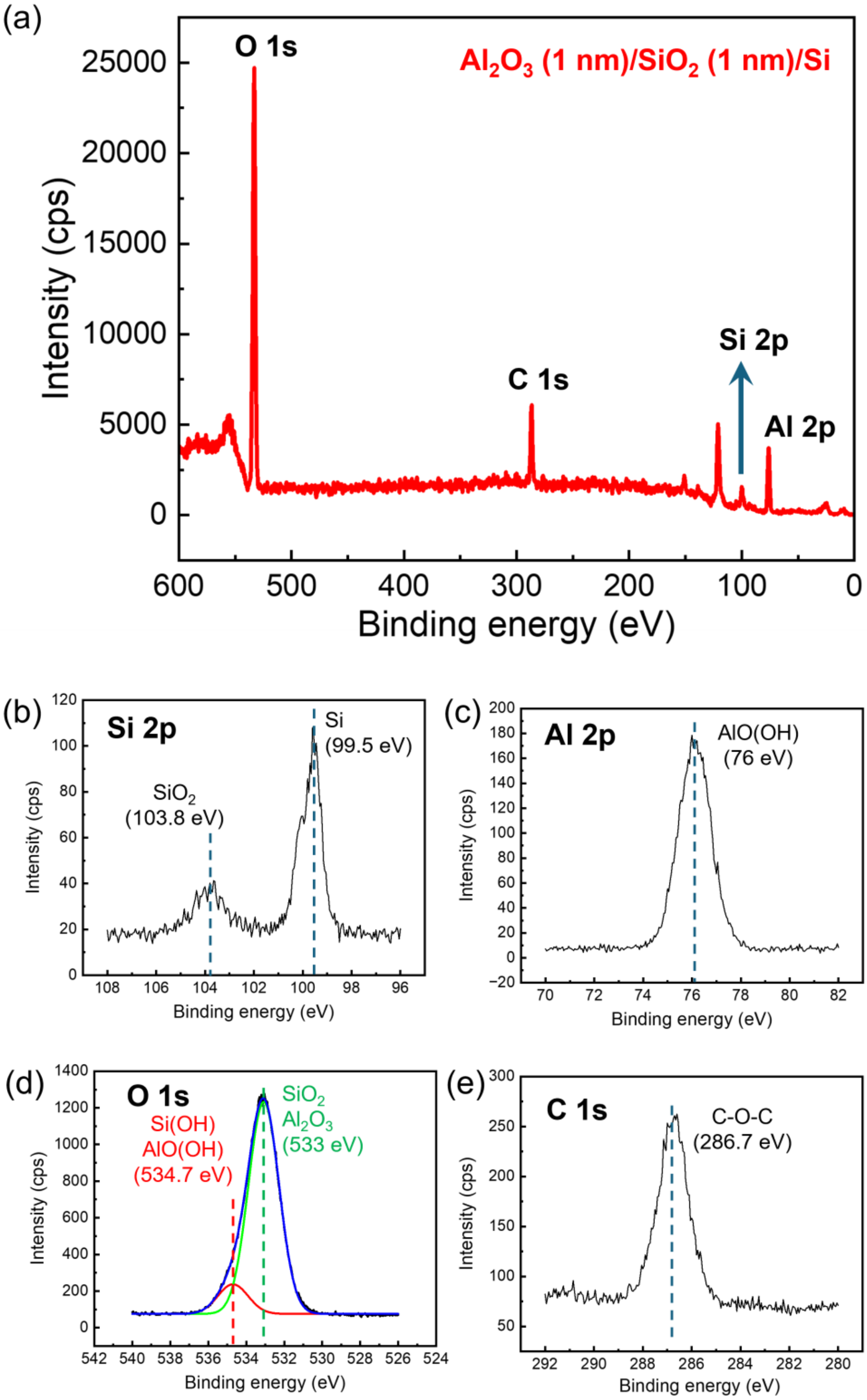

Figure S3. XPS spectra of $Al_2O_3/SiO_2/Si$ (double-tunnel junction) sample: (a) wide scan and narrow scans of (b) Si 2p, (c) Al 2p and (d) O 1s states.

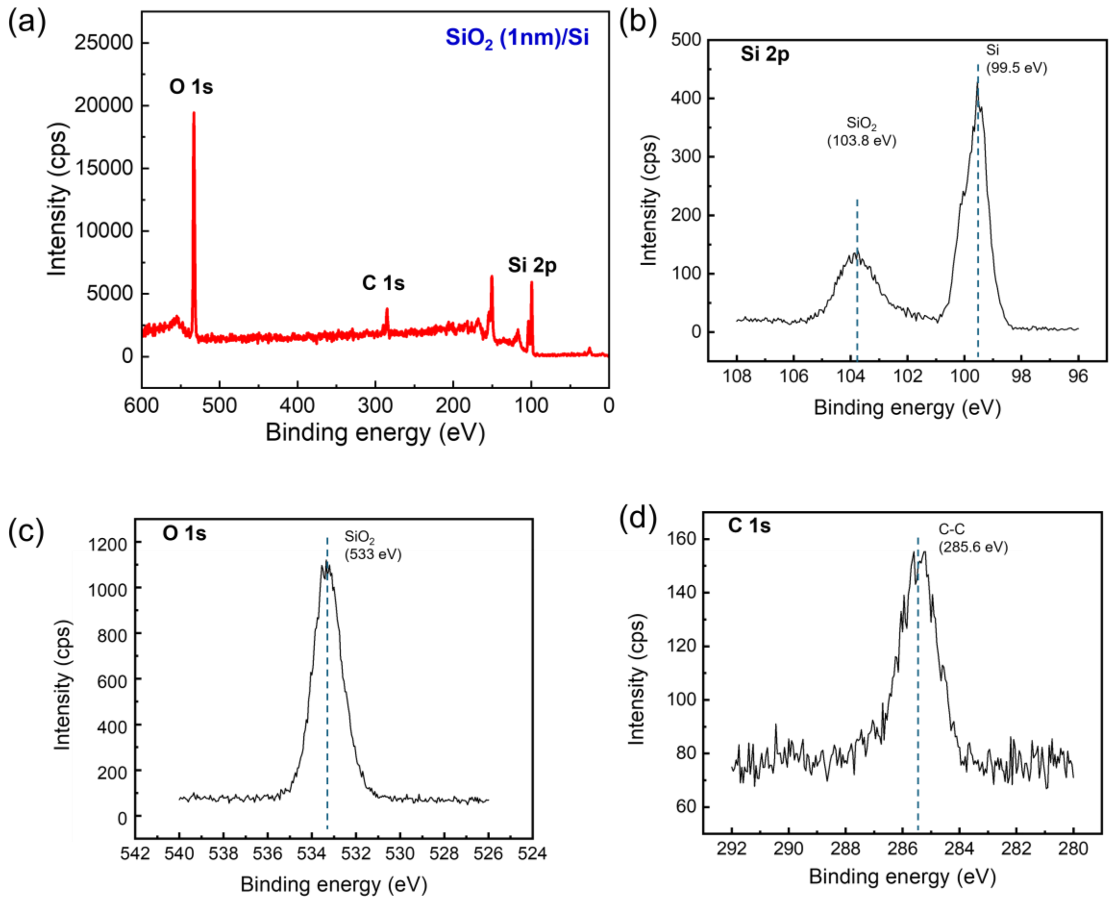

Figure S4. XPS spectra of SiO_2/Si sample: (a) wide scan and narrow scans of (b) Si 2p, (c) O 1s, and (d) C 1s states.

5. Molecular orbitals (MOs) of TEMPO-OPE in vacuum

To calculate MOs, we considered a freestanding TEMPO-OPE molecule in vacuum. The calculations were performed with Gaussian software employing Kohn-Sham density functional theory (DFT)[4] with B3LYP (Hybrid GGA with 20% HF)[5] exchange-correlation functionals with Grimme's D3 dispersion corrections (employing Becke-Johnson damping)[6] with a def2-TZVP basis set. The rotatable amide group raises *(E)-(Z)* isomerism of this molecule. **Figure 1**b (main manuscript) and **Figure S5**a show the optimized molecular structure of *(Z)*-TEMPO-OPE and *(E)*-TEMPO-OPE, respectively. The optimized molecular structures were checked by vibrational frequency calculations to make sure no imaginary mode exists. Isosurface value was 0.02 a.u. for molecular orbital plot. **Figure 1**c (main manuscript) and

Figure S5b show the molecular orbital energy diagram with corresponding MO plot for *(Z)*-TEMPO-OPE and *(E)*-TEMPO-OPE, respectively, in vacuum. **Table S1** and **S2** represents the MO plots of *(Z)*-TEMPO-OPE and *(E)*-TEMPO-OPE, respectively, in vacuum. Molecular energy level of the singly occupied molecular orbital (SOMO) is well separated from fully occupied MOs (such as HOMO, HOMO−1 etc.) and unoccupied MOs (such as LUMO, LUMO+1 etc.) for both isomers. The SOMO is mainly located at the TEMPO part, more precisely, it is nearly fully located on $\pi^*_{N\text{-}O}$ orbital in both isomers. It is evident that SOMO is singly occupied by the unpaired electron of nitroxyl group (N-O) in TEMPO-OPE molecule.

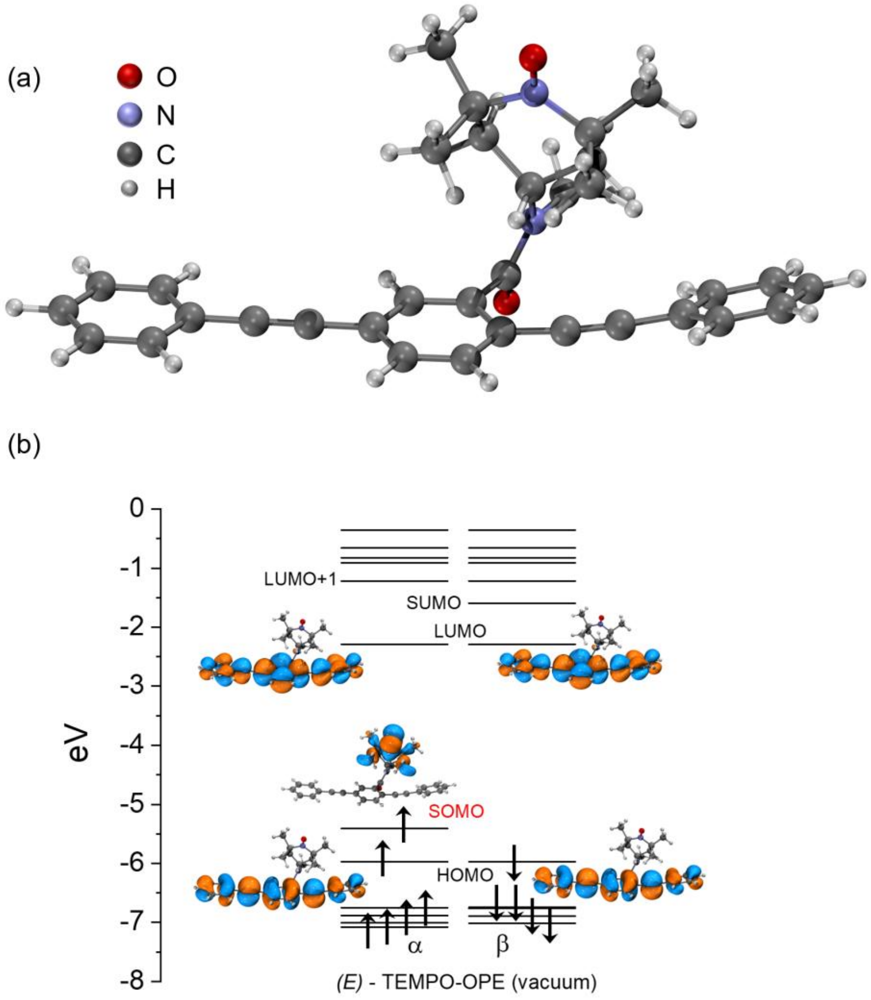

Figure S5. (a) Optimized structure of *(E)*-TEMPO-OPE in vacuum. (b) Molecular orbital energy diagram and corresponding isosurfaces for *(E)*-TEMPO-OPE in vacuum.

Table S1. Molecular-orbital isosurfaces of *(Z)*-TEMPO-OPE calculated in vacuum.

MO indices	Spin up (α)	MO indices	Spin down (β)
126 HOMO−4	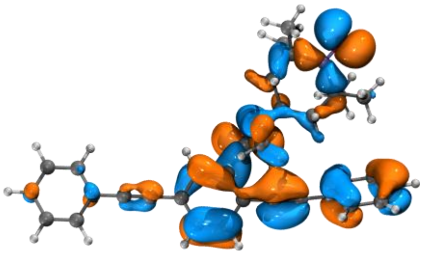	126 HOMO−4	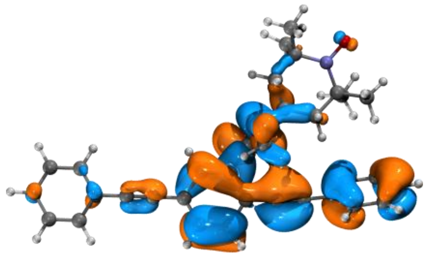
127 HOMO−3	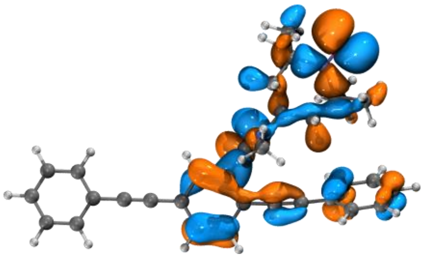	127 HOMO−3	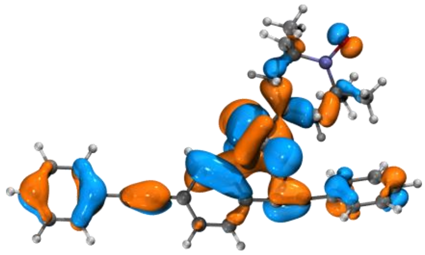
128 HOMO−2	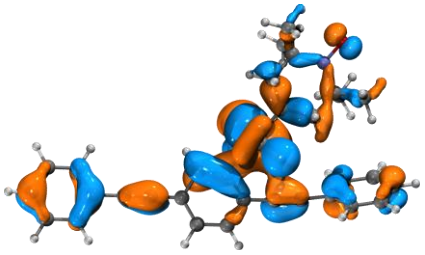	128 HOMO−2	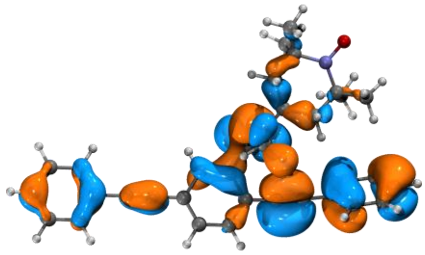
129 HOMO−1	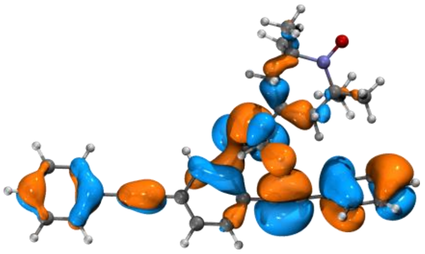	129 HOMO−1	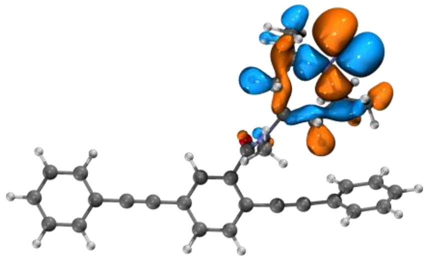
130 HOMO	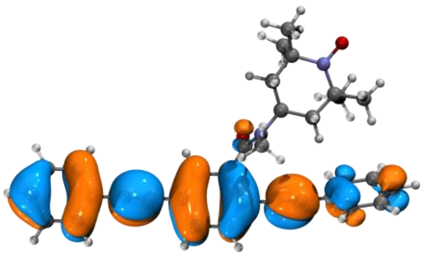	130 HOMO	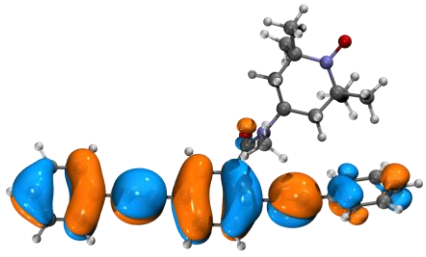

131<br>SOMO	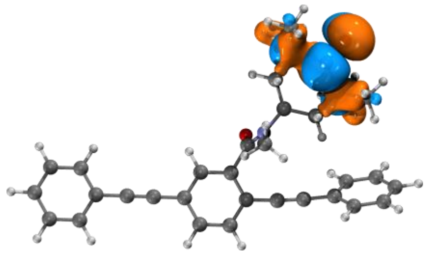	131<br>LUMO	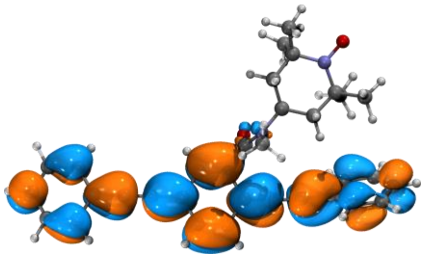
132<br>LUMO	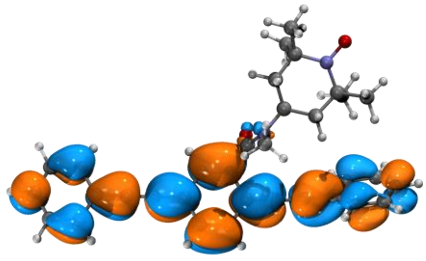	132<br>SUMO	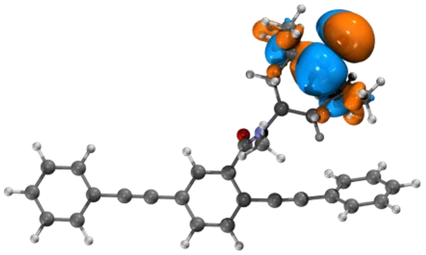
133<br>LUMO+1	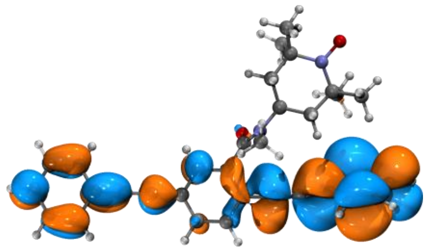	133<br>LUMO+1	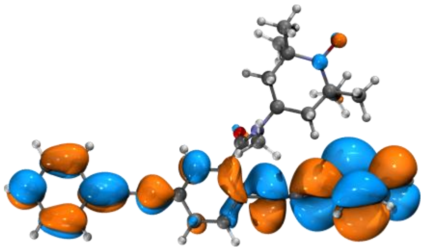
134<br>LUMO+2	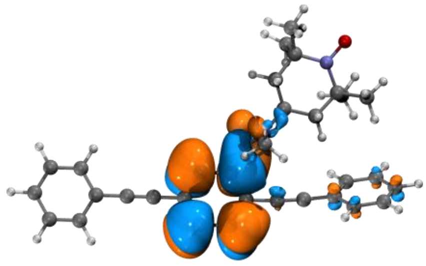	134<br>LUMO+2	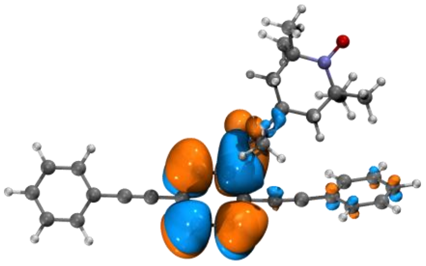

Table S2. Molecular-orbital isosurfaces of *(E)*-TEMPO-OPE calculated in vacuum.

MO indices	Spin up (α)	MO indices	Spin down (β)
126<br>HOMO−4	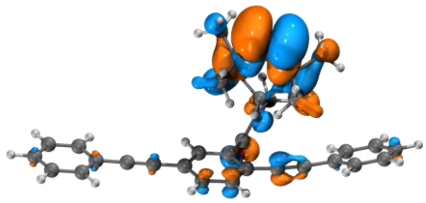	126<br>HOMO−4	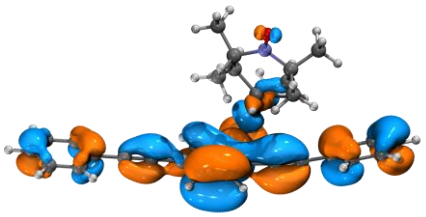

127<br>HOMO−3	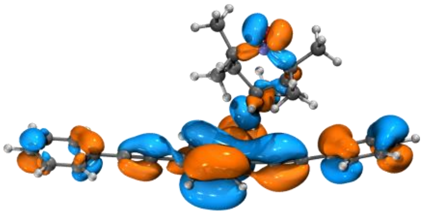	127<br>HOMO−3	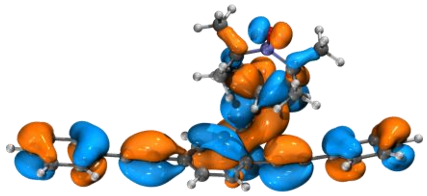
128<br>HOMO−2	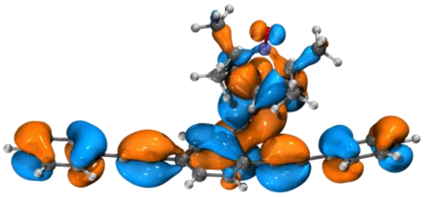	128<br>HOMO−2	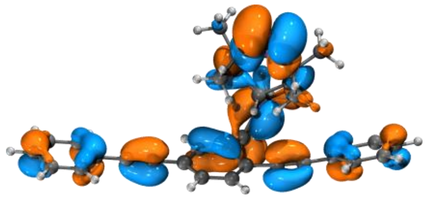
129<br>HOMO−1	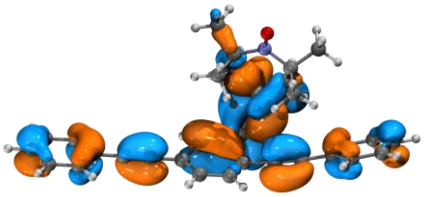	129<br>HOMO−1	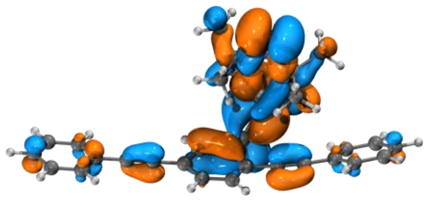
130<br>HOMO	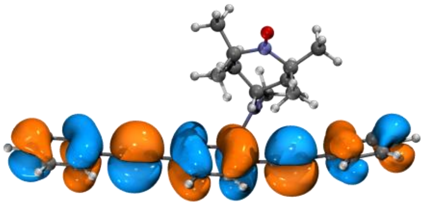	130<br>HOMO	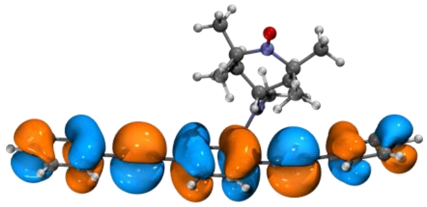
131<br>SOMO	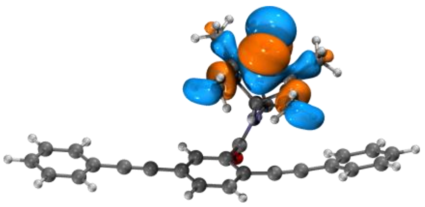	131<br>LUMO	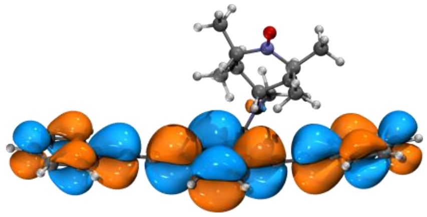
132<br>LUMO	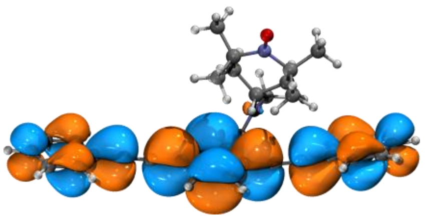	132<br>SUMO	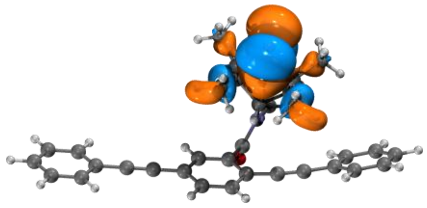
133<br>LUMO+1	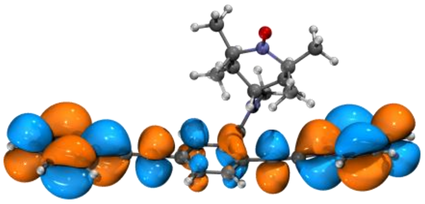	133<br>LUMO+1	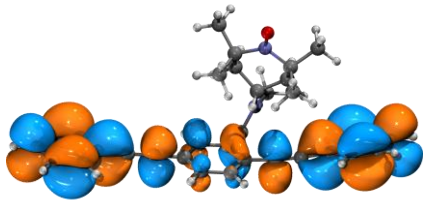

134<br>LUMO+2	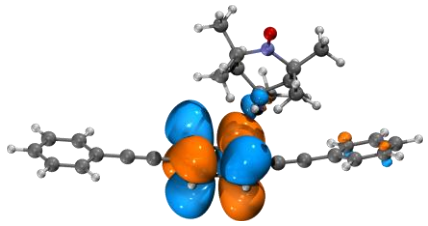	134<br>LUMO+2	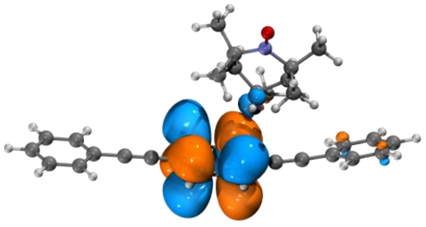

6. Vibrational spectra and structural stabilities of *(Z)*- and *(E)*-TEMPO-OPE molecules

The simulated IR and Raman spectra of the optimized *(Z)*- and *(E)*-TEMPO-OPE molecules in vacuum are shown in **Figure S6**a and **S6**b, respectively. The calculated vibrational modes of the optimized TEMPO-OPE structures in vacuum revealed only minor differences between the two conformations. The primary structural distinction between these conformations is the relative proximity of the TEMPO side chains to the OPE backbone. However, this variation mainly arises from dispersive interactions and has a negligible effect on the vibrational frequencies. Moreover, the resolution of experimentally measured IR spectra is typically much lower than the small frequency differences observed in the calculated spectra. Consequently, these spectral differences are unlikely to be resolved experimentally.

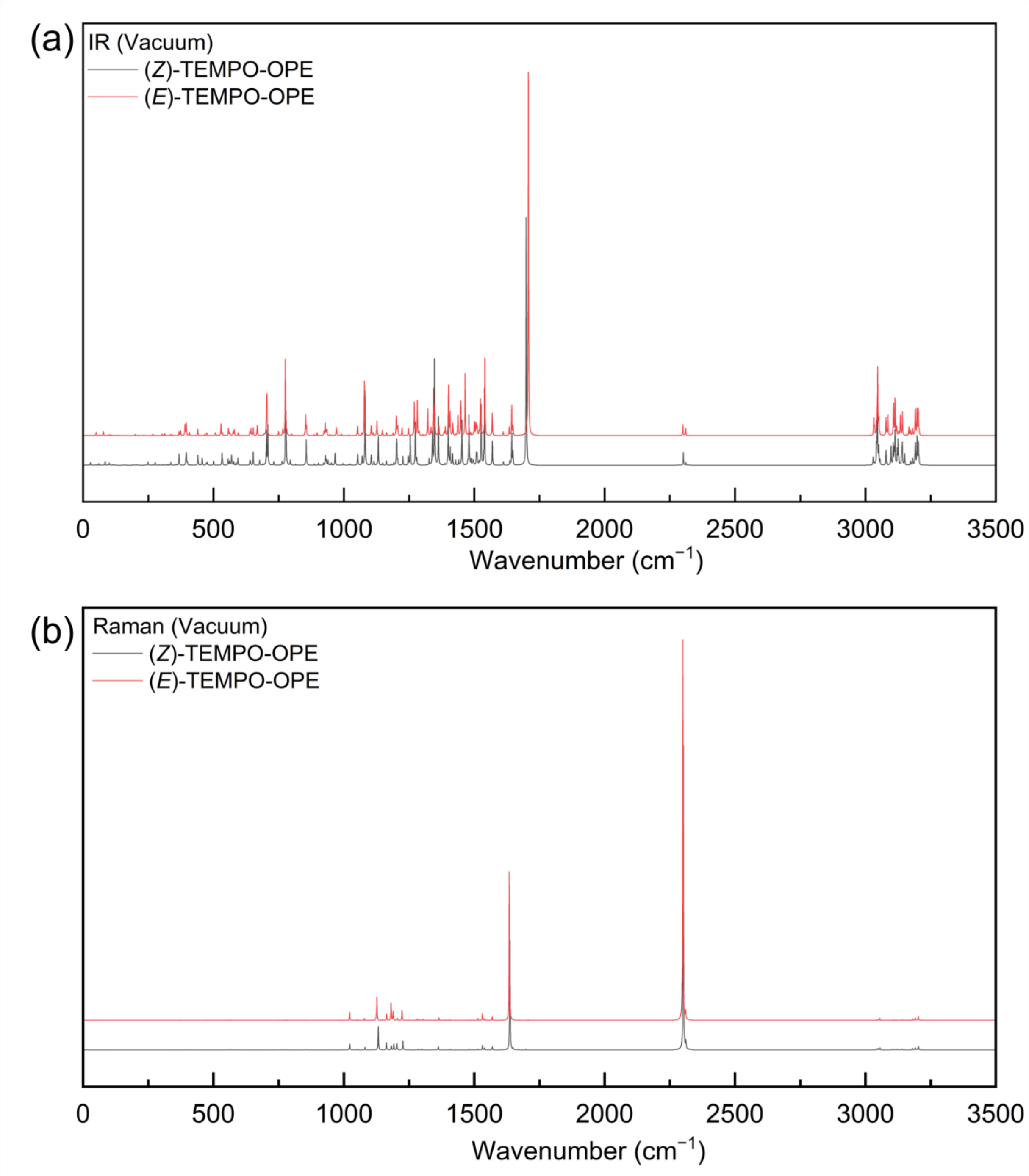

Figure S6. Simulated (a) IR and (b) Raman spectra of *(Z)*- and *(E)*-TEMPO-OPE in vacuum.

Additionally, we have calculated binding energies of the *(E)*- and *(Z)*-TEMPO-OPE on the hydro-α-SiO_2 and hydro-α-Al_2O_3 surfaces (**Table S**3). The *(Z)*-TEMPO-OPE structure is more energetically stable than the *(E)*-TEMPO-OPE structure on both the surfaces. This stability can be attributed to the fact that the molecule adopts a more flattened conformation on the surface,

resulting in a larger contact area and stronger interactions with the substrate. From an energetic perspective, the *(Z)*-TEMPO-OPE conformation seems to be more favorable.

Table S3. The binding energies of the optimized *(Z)*- and *(E)*-TEMPO-OPE molecules on the hydro-α-SiO_2 and hydro-α-Al_2O_3 surfaces.

$E_{Binding}$ (KJ/mol)	*(E)*-TEMPO-OPE	*(Z)*-TEMPO-OPE
Hydro-α-SiO_2 (100)	−156.2	−193.1
Hydro-α-Al_2O_3 (001)	−172.8	−223.5

7. DFT calculations of a double-tunnel junction incorporating TEMPO-OPE molecules

In our device TEMPO-OPE molecules are embedded between two insulating layers, namely SiO_2 and Al_2O_3. The interaction between the molecules and insulating layer's surface may change the molecule's energy level. To simulate the embedded TEMPO–OPE molecule in the double-tunnel junction, a double-layered systems (using SiO_2 and Al_2O_3) were built. Here, the hydro-α-SiO_2 (100) surface were used as the substrate layer and the hydro-α-Al_2O_3 (001) surface was used as the top layer. This is because during the ALD process for Al_2O_3 deposition, water vapor was introduced and hydroxyl (OH^-) terminated surfaces are likely to form. We also confirmed this fact from experimental viewpoint where we observed presence of hydroxyl (OH^-) group in Al_2O_3/SiO_2/Si-based double-tunnel junction using X-ray photoelectron spectroscopy (XPS) measurement (**Figure S3**). Both *(E)*- and *(Z)*-TEMPO–OPE molecules were considered in the calculations. The α-Al_2O_3 (corundum) (001) surface and α-SiO_2 (α-quartz) (100) surface were cleaved and modified from the crystal structures reported in the reference.[7] Surfaces were optimized with a fixed bottom layer. Then, molecules were docked on the relaxed surface and optimized under a single-Gamma-point k-mesh with the fixed substrate. Finally, the upper substrate layers were relaxed (keeping the bottom layer fixed) and

the system was optimized with a residual force threshold of 0.02 eV/Å for TEMPO–OPE systems and 0.01 eV/Å for others. For the double-layered structures, an additional Al_2O_3 layer was placed on the TEMPO–OPE with an appropriate distance. The periodic boundary conditions (PBC) DFT calculations were performed with the VASP 5.4.4 package within the Perdew–Burke–Ernzerhof (PBE) exchange–correlation functional[8] with Grimme's D3 dispersion corrections (Becke-Johnson damping).[6] PAW pseudopotentials and planewave basis sets with cutoff energies of 640 eV for wavefunctions were used. A 2×2×1 Monkhorst-Pack k-mesh was employed for all systems. Visualization of the systems was achieved by VMD 1.9.3.

In the main manuscript, **Figure 4**a and **4**b show the optimized structure and spin density plot of *(Z)*-TEMPO-OPE, embedded between hydroxyl (OH^-) terminated SiO_2 and Al_2O_3 layers. **Table S4** represents the MO plots of *(Z)*-TEMPO-OPE, embedded between hydroxyl (OH^-) terminated SiO_2 and Al_2O_3 layers. The orbital contribution of *(Z)*-TEMPO-OPE in the double-tunnel junction is shown in **Table S5**.

Figure S7a and **S7**b show the optimized structure and spin density plot of *(E)*-TEMPO-OPE, respectively, sandwiched between hydroxyl (OH^-) terminated SiO_2 and Al_2O_3 layers. The total density of states (DOS) of the hydro-α-Al_2O_3 / *(E)*-TEMPO-OPE / hydro-α-SiO_2 structure and partial density of states (PDOS) of *(E)*-TEMPO-OPE in the sandwiched structure are shown in **Figure S7**c and **S7**d respectively. **Table S6** represents the MO plots of *(E)*-TEMPO-OPE, embedded between hydroxyl (OH^-) terminated SiO_2 and Al_2O_3 layers. The orbital contribution of *(E)*-TEMPO-OPE in the double-tunnel junction is shown in **Table S7**.

Concerning the difference in relative orbital ordering between the PDOS of the molecules in the junction vs the isolated molecule: by comparing Figures 4d and S7d, it becomes clear that the interaction between the radical substituent and the oxide layers is different for the *(E)*- and *(Z)*-TEMPO-OPE. Accordingly, the energetics of the SOMO and SUMO located on this substituent may be affected differently (as opposed to MOs located on the backbone for which the interactions are more similar). From the junction MO plots provides

in Tables S4 and S6, one can see that the shapes of the SOMO and SUMO are also somewhat different between the isomers. For the *(Z)*-TEMPO-OPE, SOMO and SUMO seem to be relatively similar, and both have π-type interactions with the oxide layers. For the *(E)*-TEMPO-OPE, the two orbitals have different shapes, with the SOMO being more delocalized over the radical, while the SUMO is localized at the NO group in close proximity to the oxide, and interacting with it through a sigma-type arrangement. This could explain why the SUMO in the *(E)*-TEMPO-OPE is shifted compared with the isolated molecule, while for the other three cases, such a shift is much less pronounced. The difference between the *(E)*- and *(Z)*-TEMPO-OPE' PDOS illustrates the uncertainty involved in looking at a restricted set of conformations of idealized junctions rather than a full statistical sampling of realistic junction structures. This is important to keep in mind when interpreting our results: They should be understood as a qualitative guideline, rather than a fully quantitative description.

Table S4. Molecular-orbital (MO) isosurfaces of *(Z)*-TEMPO-OPE embedded between the hydroxyl (OH^{-}) terminated SiO_2 and Al_2O_3 layers.

MO indices	Spin up (α)	MO indices	Spin down (β)
1623 HOMO−2	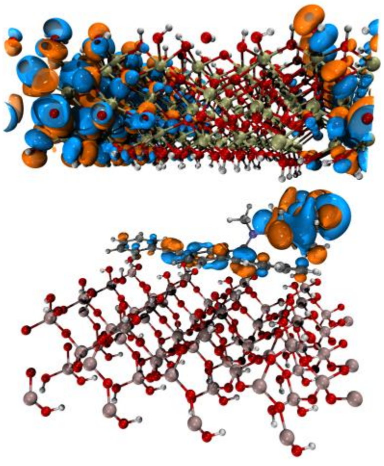	1623	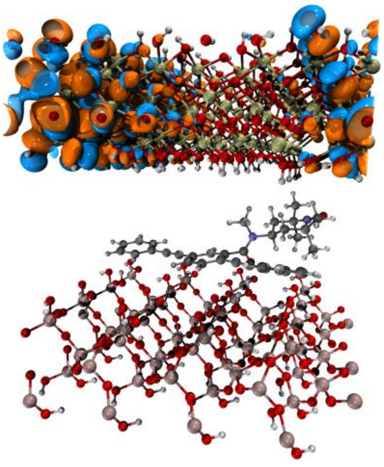

1624<br>HOMO−1	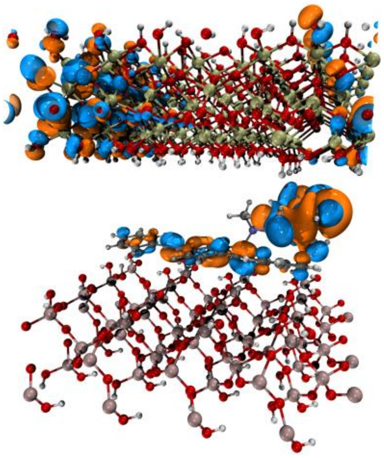	1624<br>HOMO−1	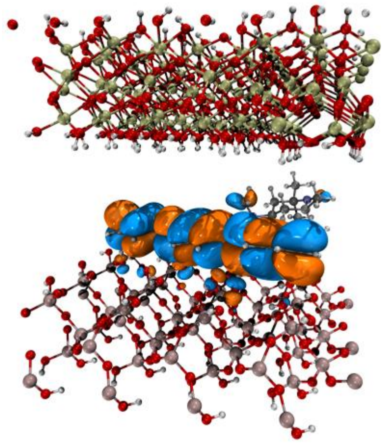
1625<br>HOMO	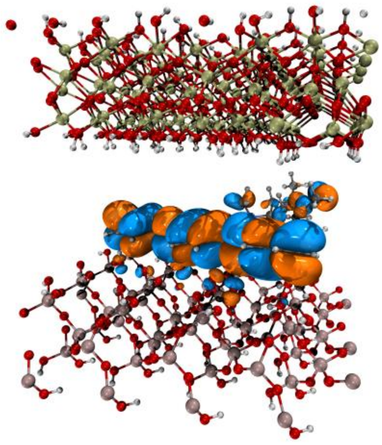	1625<br>HOMO	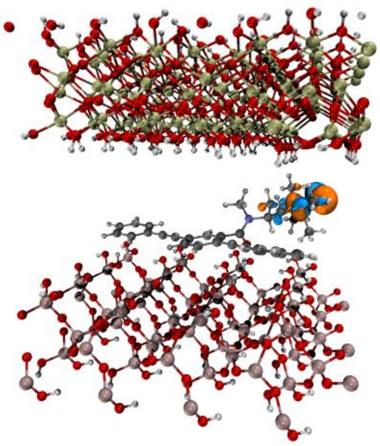
1626	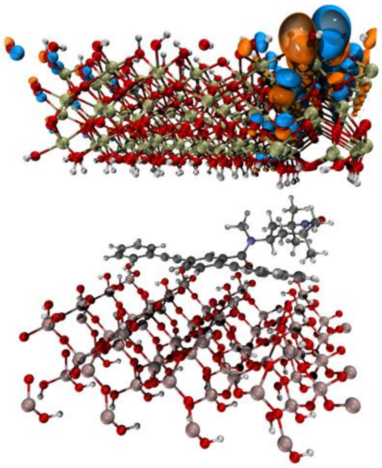	1626	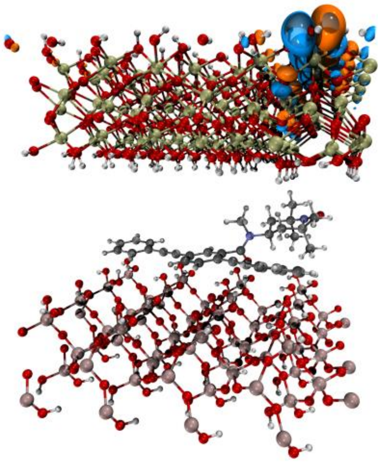

1627	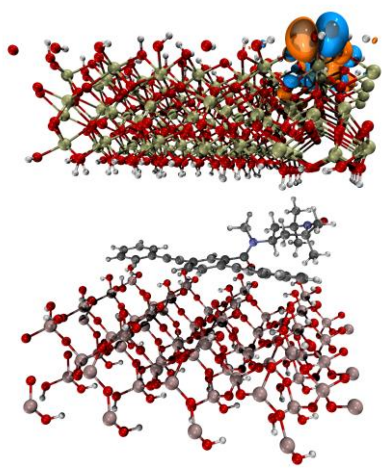	1627	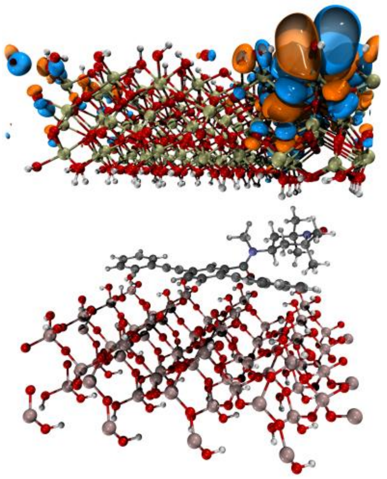
1628	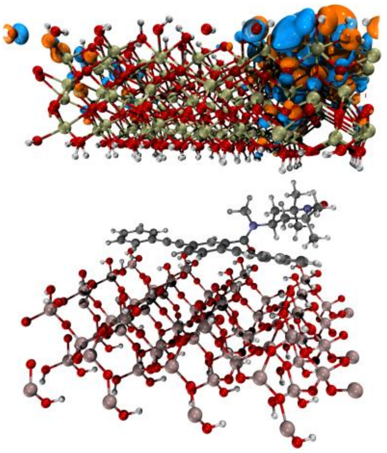	1628	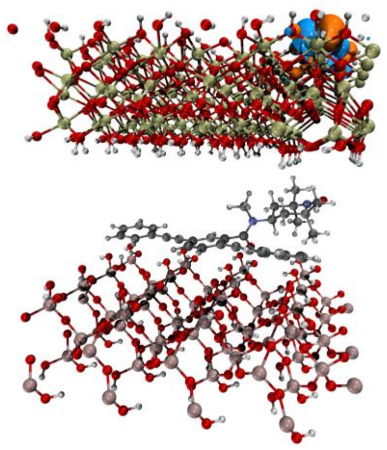
1629	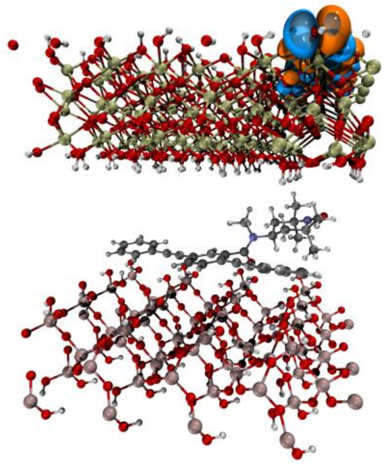	1629	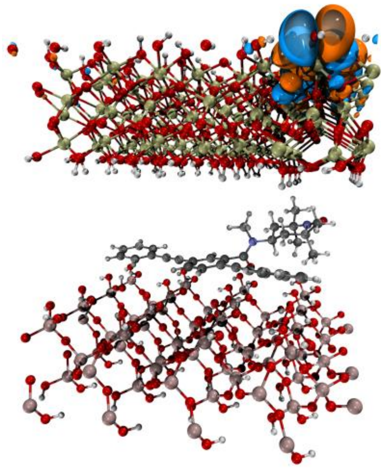

1630<br><br>SOMO	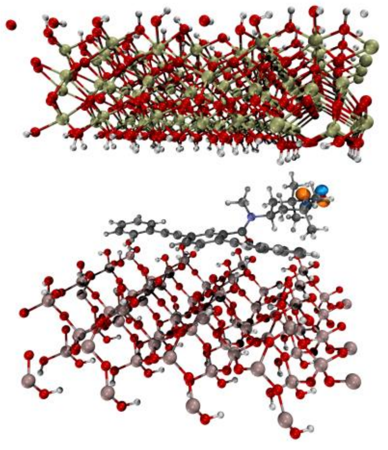	1630<br><br>LUMO	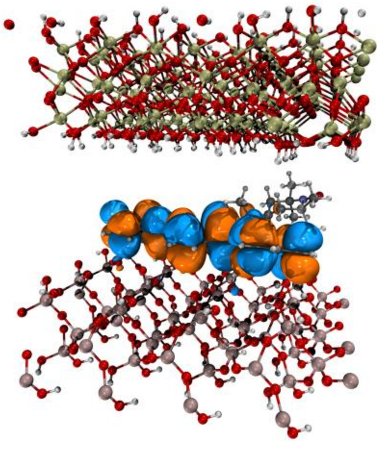
1631<br><br>LUMO	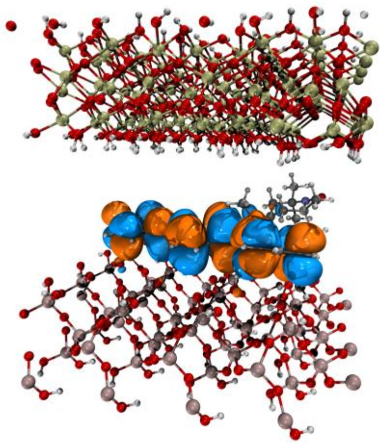	1631<br><br>SUMO	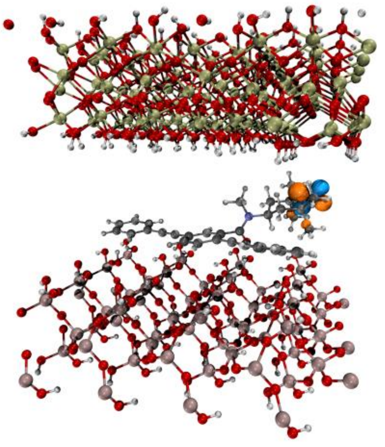
1632<br><br>LUMO+1	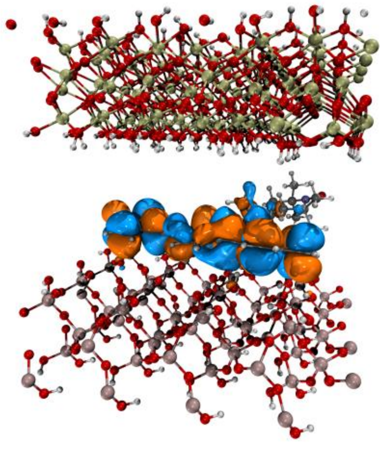	1632<br><br>LUMO+1	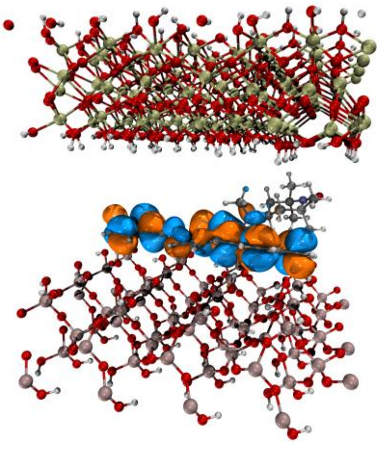

1633	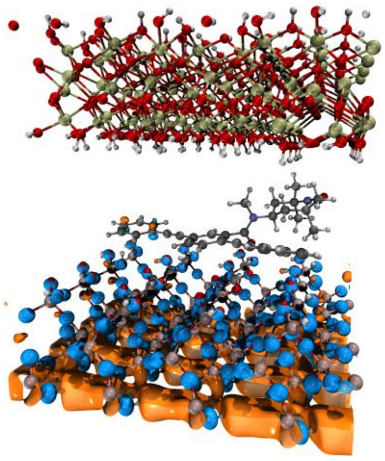	1633	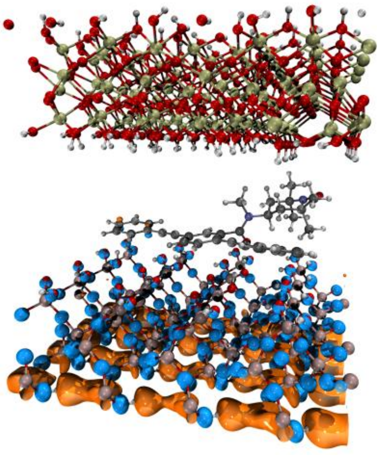

Table S5. Primary orbital contributions of *(Z)*-TEMPO-OPE embedded in the hydroxyl (OH^{-}) terminated SiO_2 and Al_2O_3 layers. The HOMO(M), SOMO(M), SUMO(M), LUMO in the table refers to the molecular orbital of *(Z)*-TEMPO-OPE in vacuum.

spin (up)			
**Index**	$E\text{-}E_f$ (eV)	site	MO Contribution
**1622**	−1.631	Al_2O_3	
**1623**	−1.601	Al_2O_3+TEMPO+OPE	HOMO(M) + HOMO-3(M) + MO(Al_2O_3)
**1624**	−1.599	Al_2O_3+TEMPO+OPE	HOMO(M) + HOMO-3(M) − MO(Al_2O_3)
**1625**	−1.556	TEMPO+OPE	HOMO(M) − HOMO-3(M)
**1626**	−1.212	Al_2O_3	
**1627**	−1.149	Al_2O_3	
**1628**	−1.061	Al_2O_3	
**1629**	−0.996	Al_2O_3	
**1630**	−0.091	TEMPO	SOMO(M)
**1631**	0.911	OPE	LUMO(M)

**1632**	1.835	OPE	LUMO+1(M)
**1633**	1.989	SiO_2	
spin (down)			
**Index**	$E-E_f$ (eV)	site	MO Contribution
**1622**	−1.631	Al_2O_3	
**1623**	−1.601	Al_2O_3	
**1624**	−1.557	OPE	HOMO(M)
**1625**	−1.335	TEMPO	HOMO-1(M)
**1626**	−1.212	Al_2O_3	
**1627**	−1.149	Al_2O_3	
**1628**	−1.061	Al_2O_3	
**1629**	−0.996	Al_2O_3	
**1630**	0.911	OPE	LUMO(M)
**1631**	1.348	TEMPO	SUMO(M)
**1632**	1.836	OPE	LUMO+2(M)
**1633**	1.989	SiO_2	

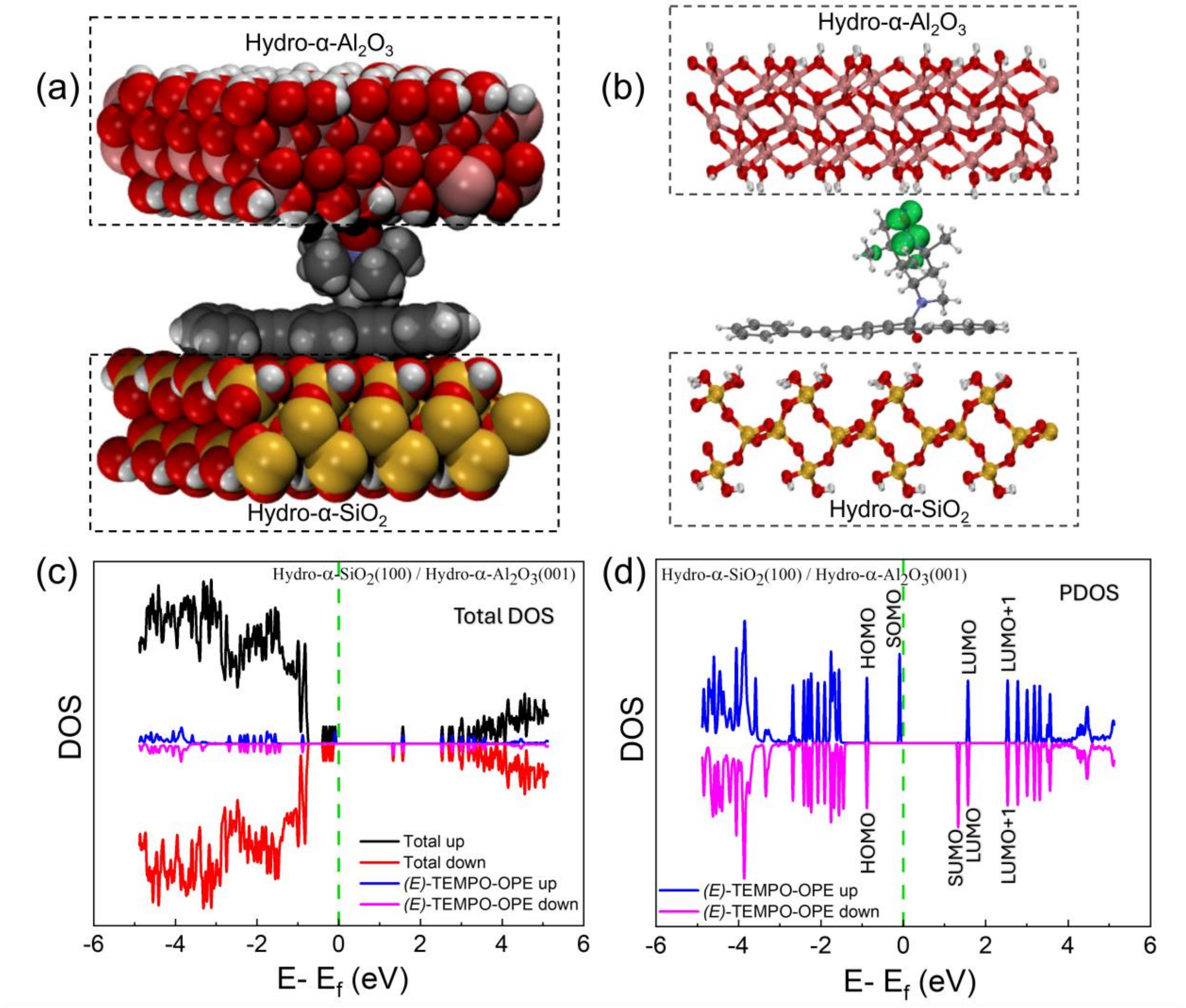

Figure S7. (a) Optimized structure, (b) spin density isosurfaces, (c) total DOS, and (d) partial DOS of *(E)*-TEMPO-OPE embedded between the hydro-α-SiO_2 and the hydro-α-Al_2O_3 layers. The Fermi level is shown by green dashed line in DOS plots.

Table S6. Molecular-orbital (MO) isosurfaces of *(E)*-TEMPO-OPE embedded between the hydroxyl (OH^-) terminated SiO_2 and Al_2O_3 layers.

MO indices	Spin up (α)	MO indices	Spin down (β)
1610	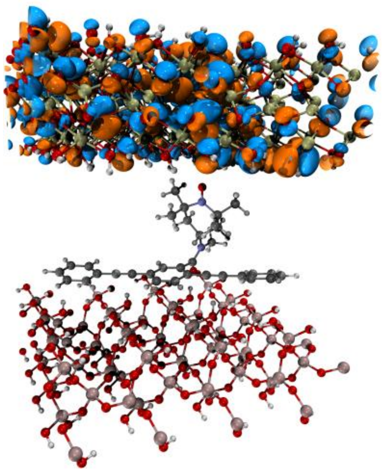	1610	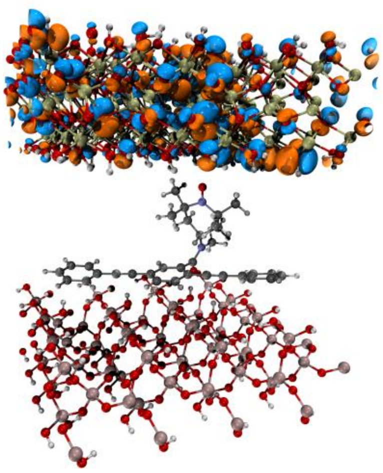
1611	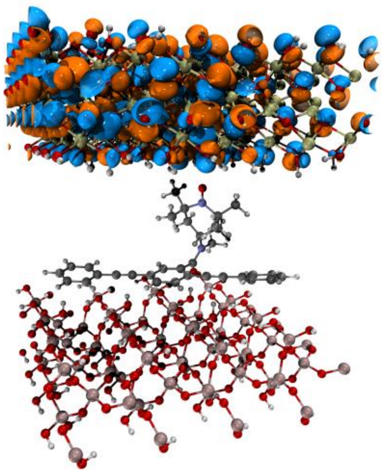	1611	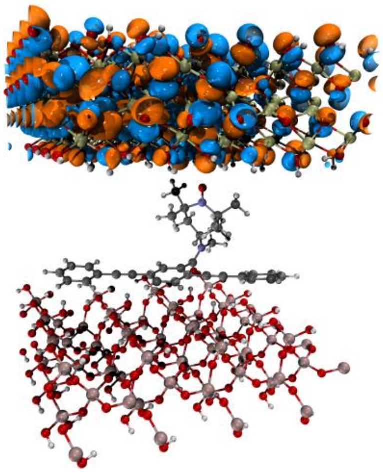
1612 HOMO	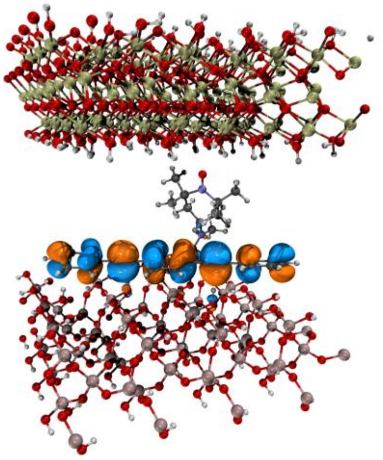	1612 HOMO	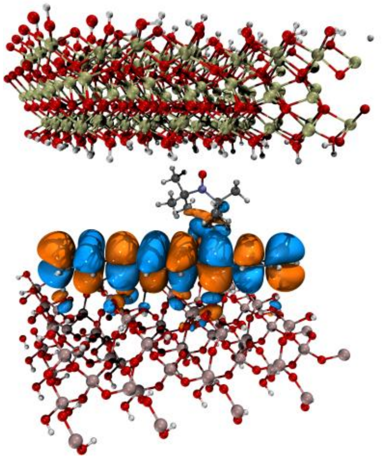

1613	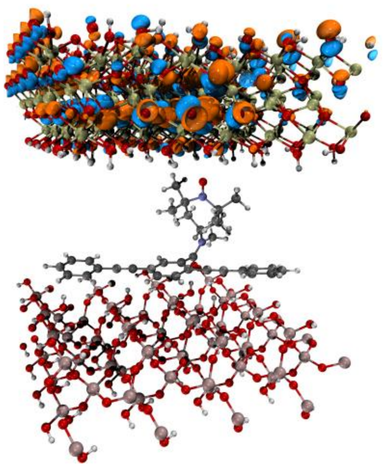	1613	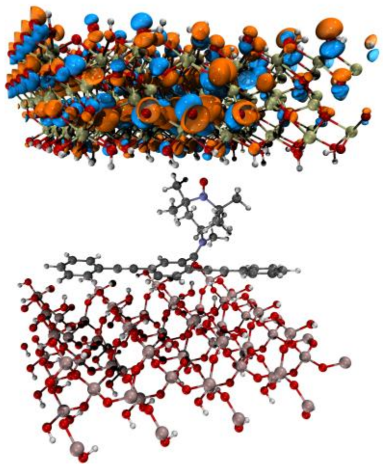
1614	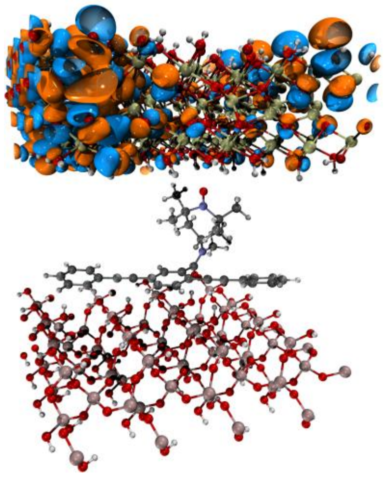	1614	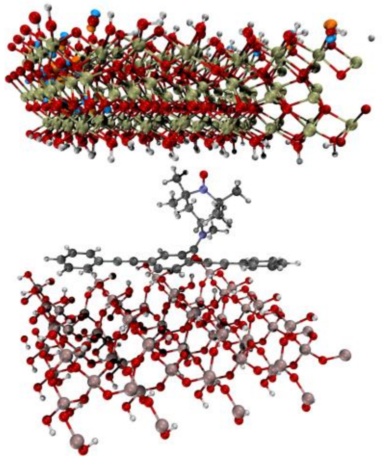
1615	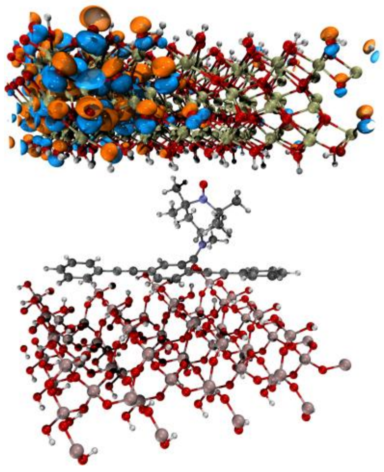	1615	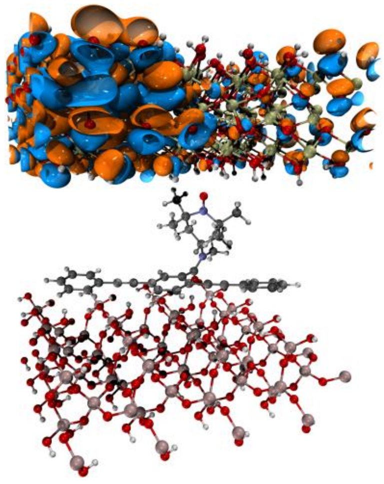

1616	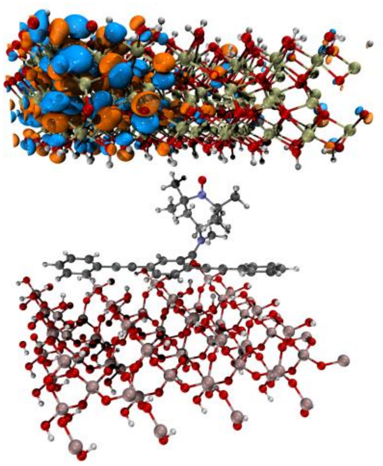	1616	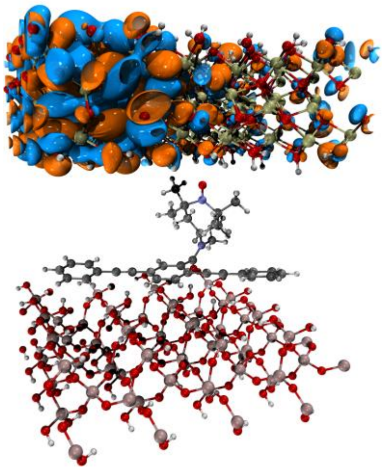
1617	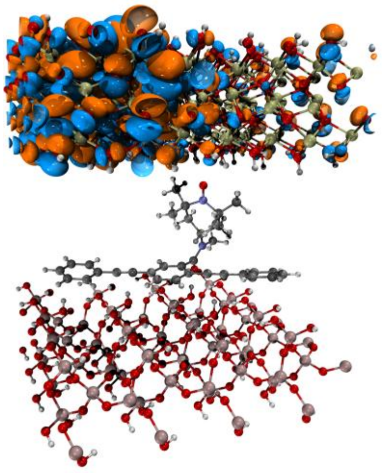	1617	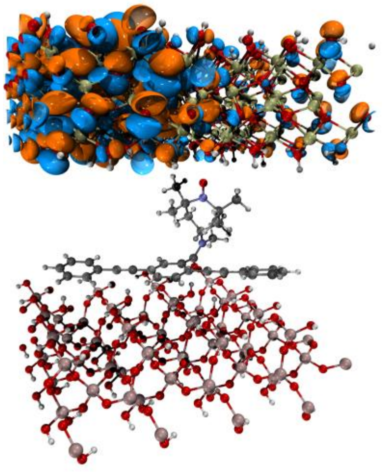
1618	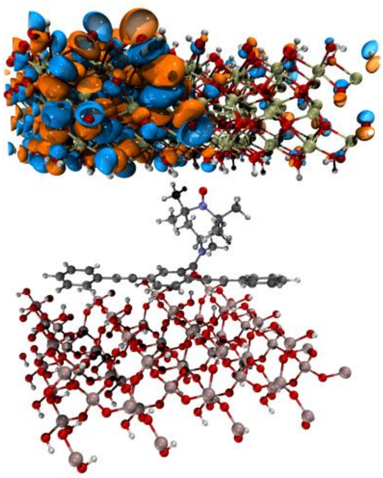	1618	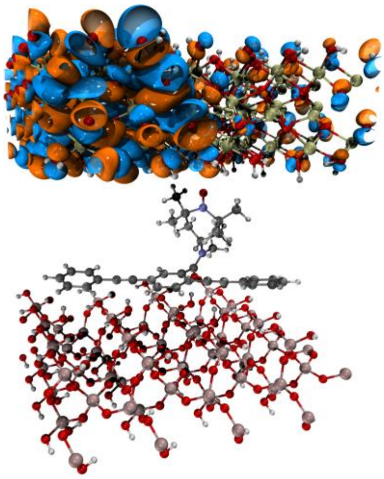

1619	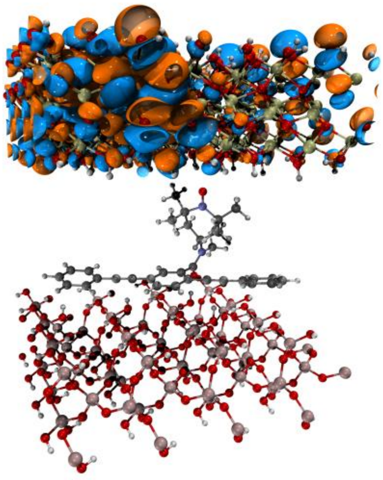	1619	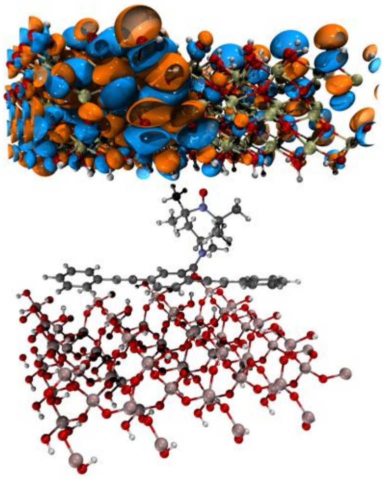
1620	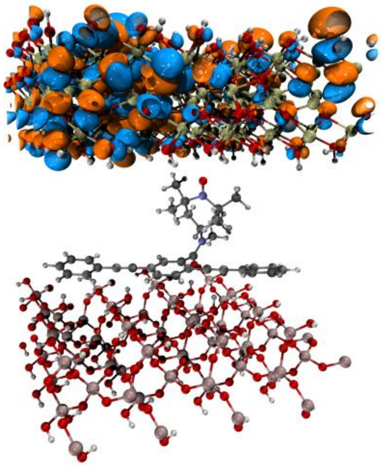	1620	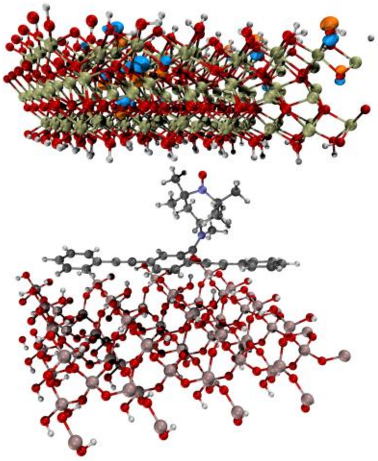
1621	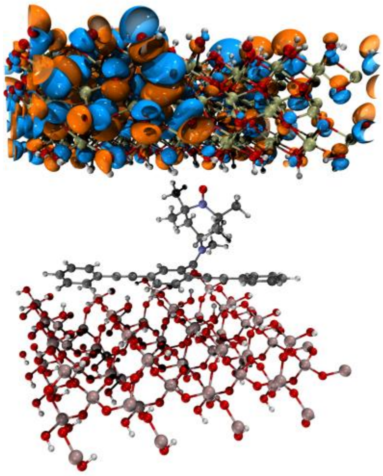	1621	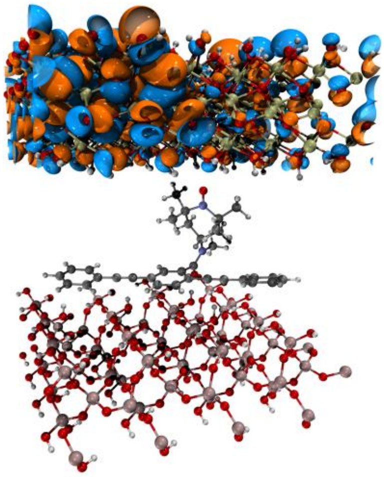

1622	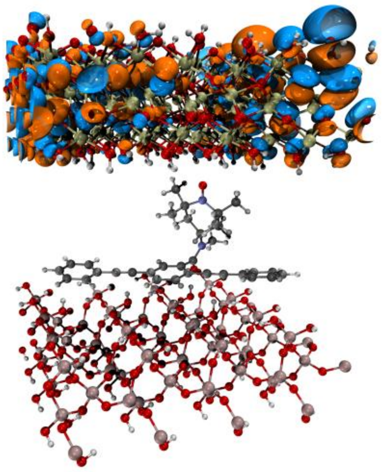	1622	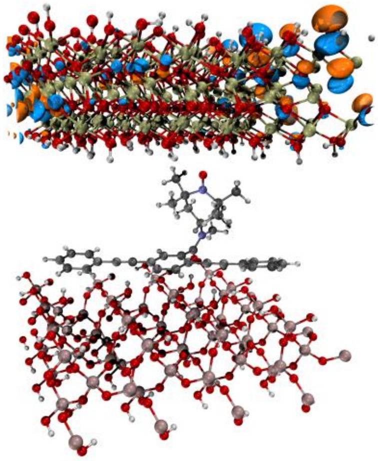
1623	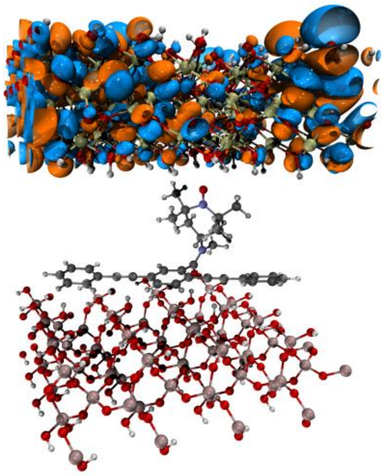	1623	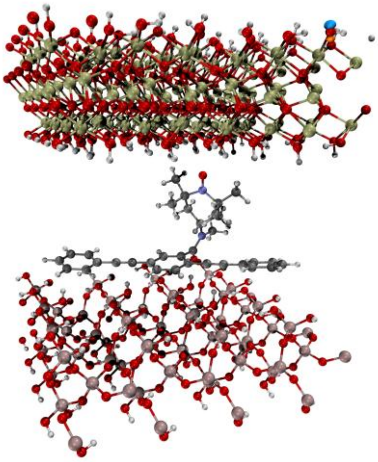
1624	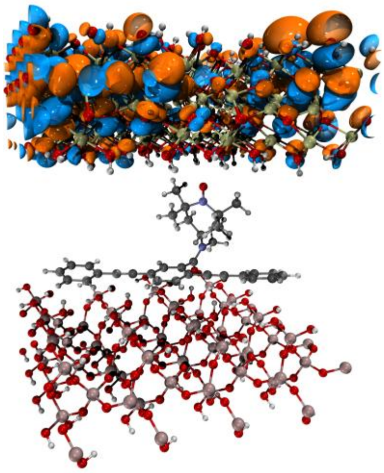	1624	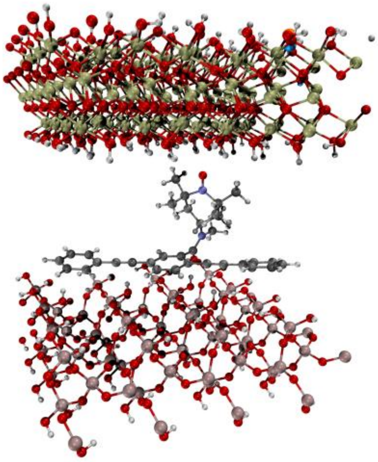

1625	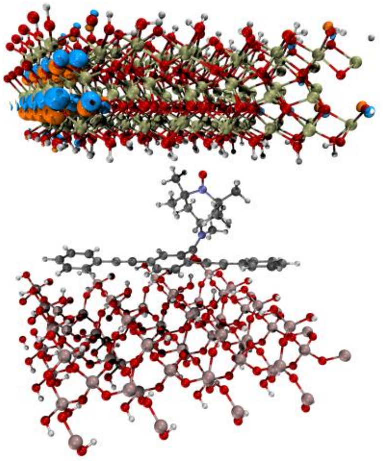	1625	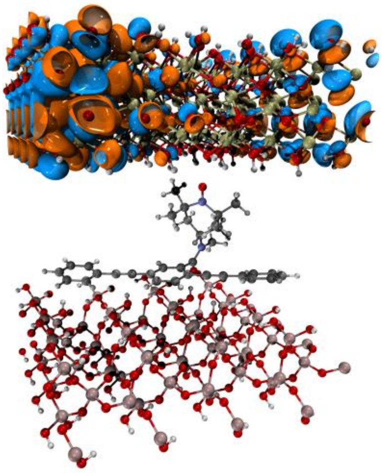
1626	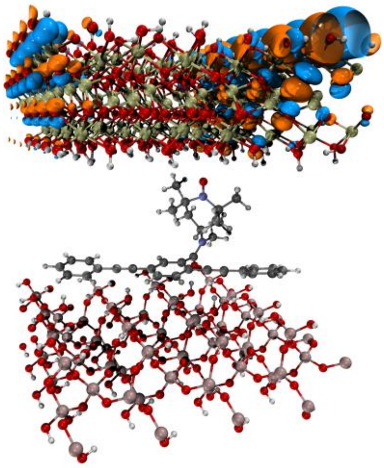	1626	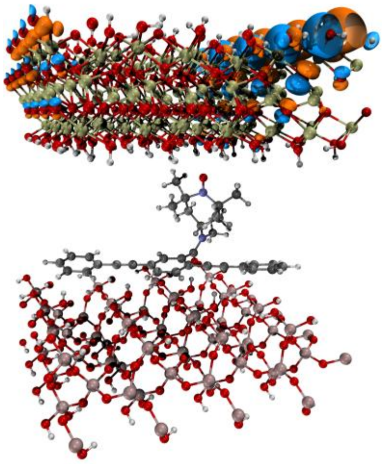
1627	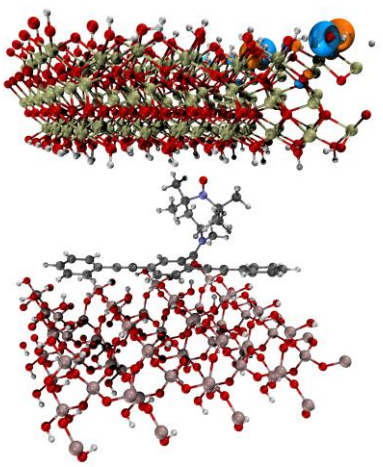	1627	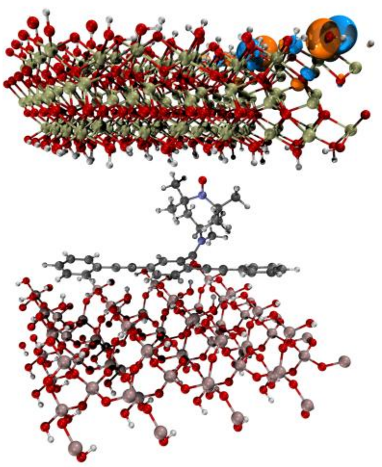

<table>
<tr><td>1628</td><td>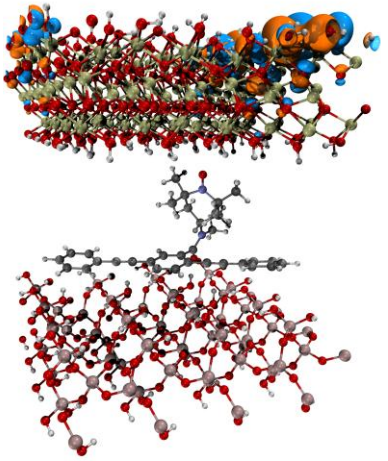</td><td>1628</td><td>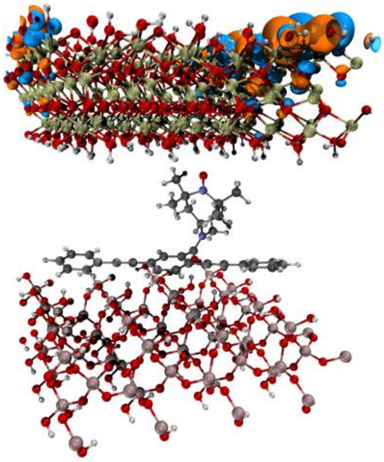</td></tr>
<tr><td>1629</td><td>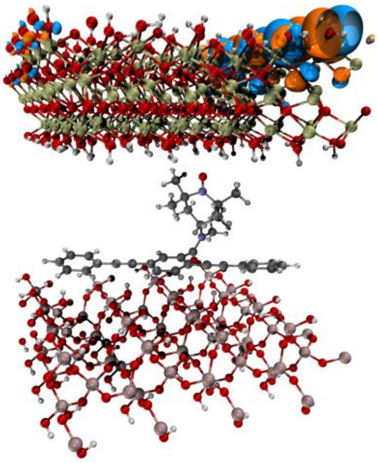</td><td>1629</td><td>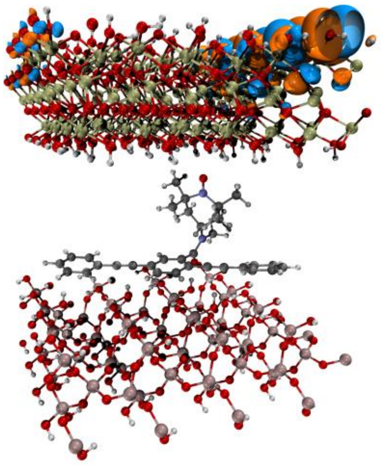</td></tr>
<tr><td>1630
SOMO</td><td>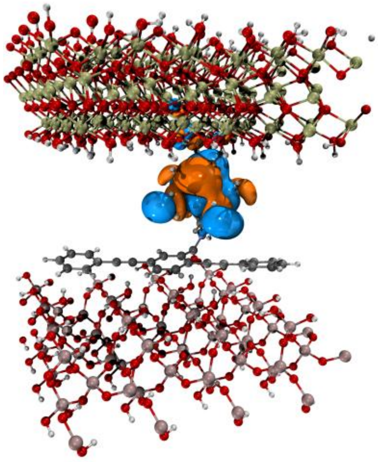</td><td>1630
SUMO</td><td>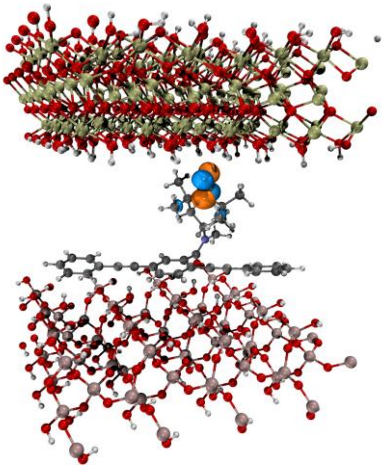</td></tr>
</table>

1631<br>LUMO	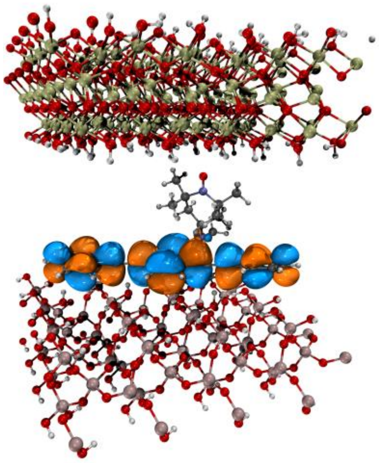	1631<br>LUMO	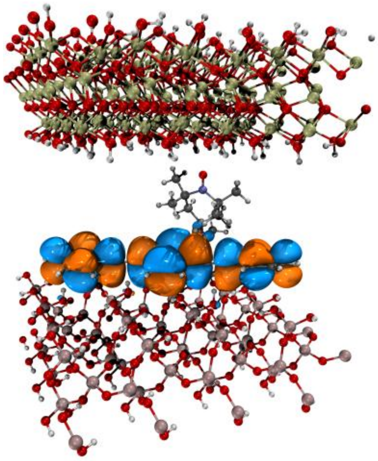
1632<br>LUMO+1	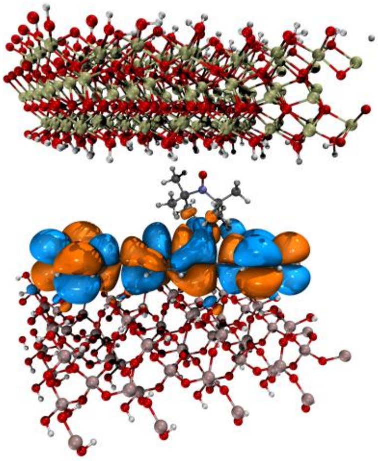	1632<br>LUMO+1	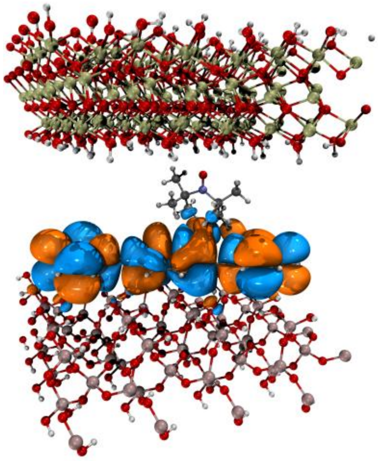
1633	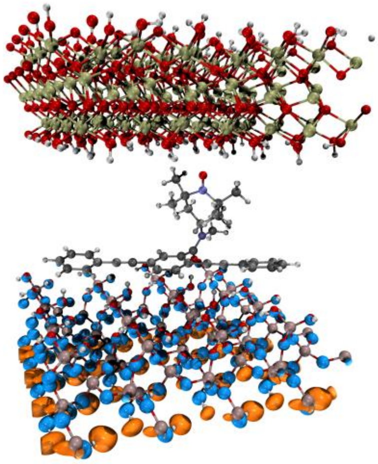	1633	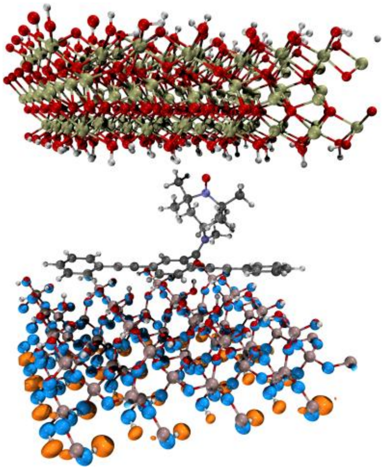

Table S7. Primary orbital contributions of *(E)*-TEMPO-OPE embedded in the hydroxyl (OH^-) terminated SiO_2 and Al_2O_3 layers. The HOMO(M), SOMO(M), SUMO(M), LUMO in the table refers to the molecular orbital of *(E)*-TEMPO-OPE in vacuum.

spin (up)			
**Index**	**E−E$_f$ (eV)**	**site**	**MO Contribution**
**1610**	−0.9695	Al_2O_3	
**1611**	−0.9019	Al_2O_3	
**1612**	−0.8883	OPE	HOMO(M)
**1613**	−0.8780	Al_2O_3	
**1614**	−0.8700	Al_2O_3	
**1615**	−0.8633	Al_2O_3	
**1616**	−0.8631	Al_2O_3	
**1617**	−0.8502	Al_2O_3	
**1618**	−0.8501	Al_2O_3	
**1619**	−0.8304	Al_2O_3	
**1620**	−0.8289	Al_2O_3	
**1621**	−0.8179	Al_2O_3	
**1622**	−0.8160	Al_2O_3	
**1623**	−0.8128	Al_2O_3	
**1624**	−0.8086	Al_2O_3	
**1625**	−0.7789	Al_2O_3	
**1626**	−0.3867	Al_2O_3	
**1627**	−0.2704	Al_2O_3	
**1628**	−0.2690	Al_2O_3	
**1629**	−0.1513	Al_2O_3	

**1630**	−0.0950	TEMPO	SOMO(M)
**1631**	1.5646	OPE	LUMO(M)
**1632**	2.5267	OPE	LUMO+1(M)
**1633**	2.6339	SiO_2	
spin (down)			
**Index**	**E−E_f (eV)**	**site**	**MO Contribution**
**1610**	−0.9694	Al_2O_3	
**1611**	−0.9017	Al_2O_3	
**1612**	−0.8883	OPE	HOMO(M)
**1613**	−0.8779	Al_2O_3	
**1614**	−0.8700	Al_2O_3	
**1615**	−0.8633	Al_2O_3	
**1616**	−0.8631	Al_2O_3	
**1617**	−0.8502	Al_2O_3	
**1618**	−0.8500	Al_2O_3	
**1619**	−0.8304	Al_2O_3	
**1620**	−0.8289	Al_2O_3	
**1621**	−0.8179	Al_2O_3	
**1622**	−0.8160	Al_2O_3	
**1623**	−0.8128	Al_2O_3	
**1624**	−0.8085	Al_2O_3	
**1625**	−0.7789	Al_2O_3	
**1626**	−0.3867	Al_2O_3	

**1627**	−0.2704	Al_2O_3	
**1628**	−0.2690	Al_2O_3	
**1629**	−0.1513	Al_2O_3	
**1630**	1.3278	TEMPO	SUMO(M)
**1631**	1.5648	OPE	LUMO(M)
**1632**	2.5268	OPE	LUMO+1(M)
**1633**	2.6339	SiO_2	

8. Effect of external magnetic field on the *I*-*V* and d*I*/d*V* curves of non-radical OPE samples without TEMPO group

Figure S8 shows the device structure of a Si-based double-tunnel junction incorporating non-radical OPE molecules. OPE molecules have closed-shell systems without any unpaired electron. The *I*-*V* and d*I*/d*V* curves of this device without magnetic field are presented in **Figure S9**a and **S9**b, respectively. Clear staircases in *I*-*V* curve and prominent d*I*/d*V* peaks were observed. The peak observed at −0.69 V in d*I*/d*V* curve can be assigned as HOMO energy level, whereas the peak seen at 0.70 V can be assigned as LUMO energy level of OPE molecules. The mean value of HOMO d*I*/d*V* peak was estimated to be −0.70 ± 0.07 V, while that of LUMO d*I*/d*V* peak was 0.68 ± 0.05 V (**Figure S10**d). **Figure S9**c and **S9**d depict the *I*-*V* and d*I*/d*V* curves of the OPE device under magnetic field ranging from 0 T to 7 T. No significant changes were observed in the *I*-*V* and d*I*/d*V* curves of the OPE sample under magnetic field. **Figure S9**e and **S9**f show the MR curves of the OPE sample at a voltage of −0.82 V (near HOMO energy level of OPE) and 0.75 V (near LUMO energy level of OPE), respectively. No significant MRs were observed in the OPE samples. The temperature was fixed at 3 K during all the measurements. These results indicate that closed-shell system OPE do not have magnetic properties in double-tunnel junction.

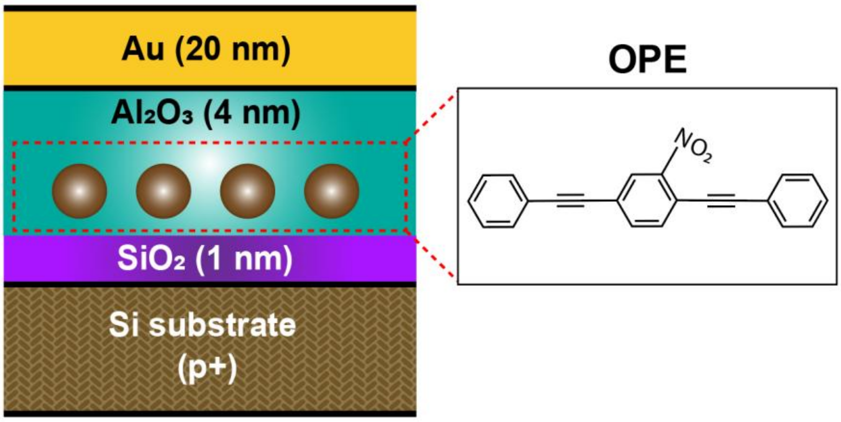

Figure S8. Schematic cross-section of double-tunnel junction incorporating non-radical OPE molecules without TEMPO group.

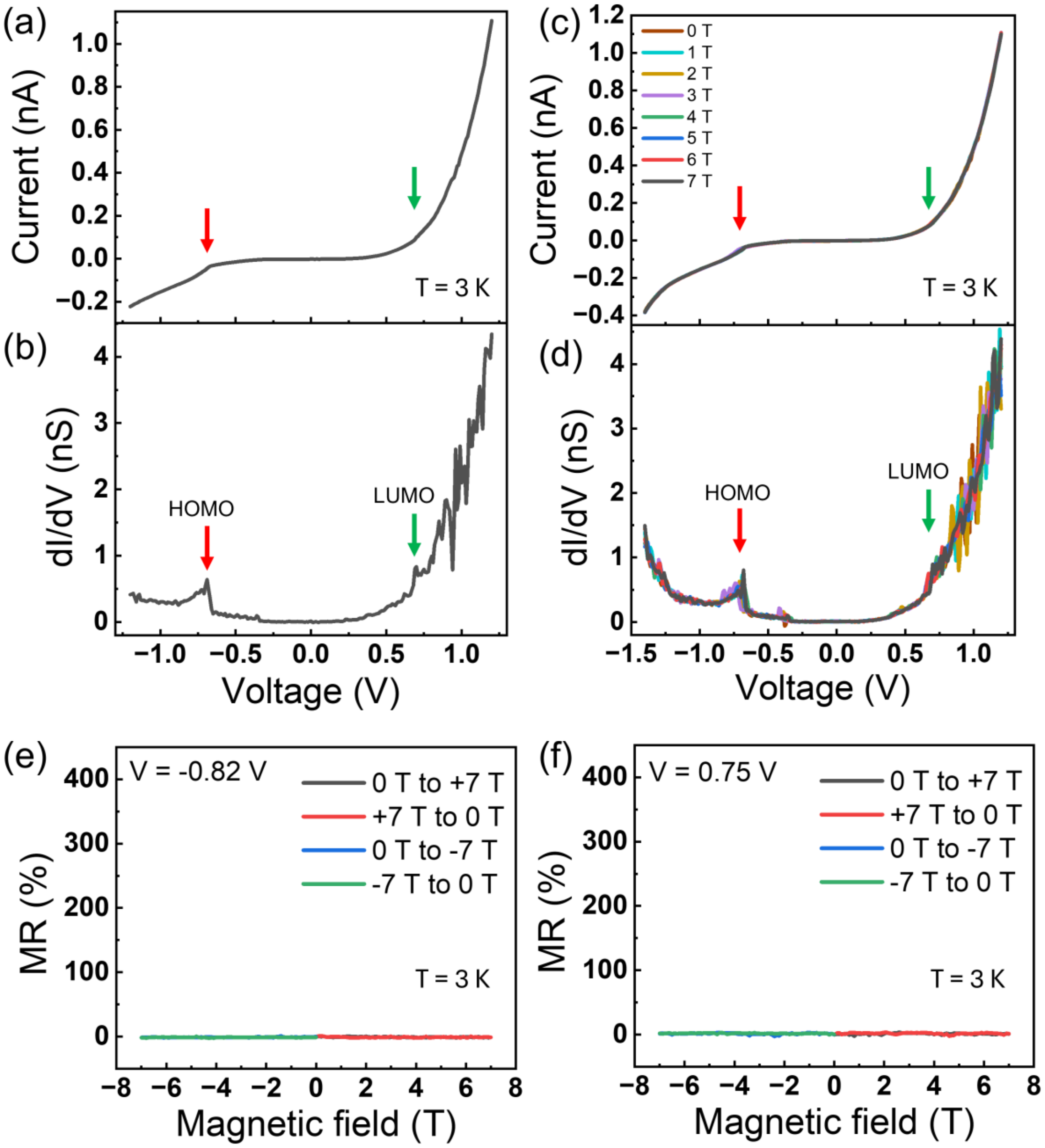

Figure S9. (a) *I*-*V* and (b) d*I*/d*V* curves of non-radical OPE embedded in a Si-based double-tunnel junction without magnetic field. Magnetic field dependence of (c) *I*-*V* and (d) d*I*/d*V* curves of the same sample. MR curves were measured (e) at −0.82 V (near HOMO energy level of OPE) and (f) at 0.75 V (near LUMO energy level of OPE) in the same sample. For (a)–(f) measurement temperature was fixed at 3 K.

9. Histograms of the tunneling currents and d*I*/d*V* peak positions corresponding to the HOMO and LUMO in TEMPO-OPE and non-radical OPE devices

For the TEMPO-OPE samples, a total of 119 devices were measured, of which 25 devices exhibited d*I*/d*V* curves reflecting molecular orbitals. The yield was estimated to be 21 %. The histogram plots of the tunneling current associated with HOMO and LUMO are shown in **Figure S10**a and **S10**b, respectively. The tunneling current varies within the range between 1 to 20 pA. The statistical distribution plots of the HOMO and LUMO d*I*/d*V* peaks in TEMPO-OPE devices is shown in **Figure S10**c. The mean values of d*I*/d*V* peak positions were estimated to be −0.68 ± 0.12 V for the HOMO and 1.08 ± 0.09 V for the LUMO, respectively, resulting in the HOMO–LUMO gap of 1.8 V.

For the reference non-radical OPE (without TEMPO group) samples, clear d*I*/d*V* curves were observed in 40 out of a total 253 devices, corresponding to a device yield of 16 %. The statistical distribution plots of the HOMO and LUMO d*I*/d*V* peaks for the non-radical OPE devices are shown in **Figure S10**d. The mean value d*I*/d*V* peak positions were −0.70 ± 0.07 V for the HOMO and 0.68 ± 0.05 V for the LUMO, leading to the HOMO–LUMO gap of 1.4 V.

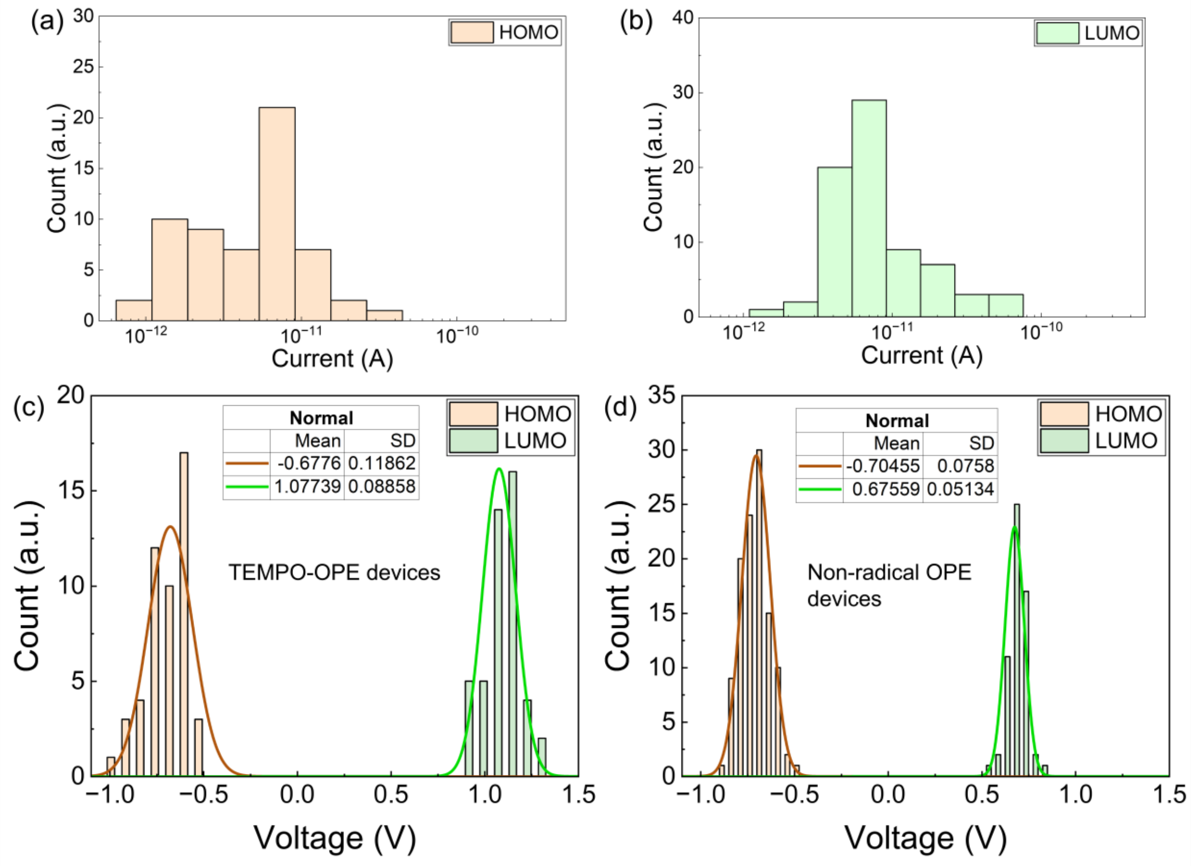

Figure S10. Histogram plots of the tunneling current associated with (a) HOMO and (b)LUMO of the TEMPO-OPE devices. The statistical distribution of the HOMO and LUMO peak positions for (c) TEMPO-OPE and (d) non-radical OPE devices.

Additionally, it is noted that the experimentally estimated HOMO−LUMO gap of TEMPO-OPE was 1.8 eV, while the calculated value is 2.5 eV (**Figure 4**d and **Figure S7**d). This difference would be caused by the assumption of the ideal junction configurations in the DFT calculations.

I. It is measured for molecules interacting with the oxide layers and possibly with other molecules, while the calculated data shown in **Figure 4**d in the manuscript look at one plausible configuration of a single molecule in an idealized oxide tunnel junction. In the measured device, the molecules are embedded in the aluminum oxide layer. By contrast, in our calculations, we have assumed an idealized junction configuration, in which a single molecule is located at the interface between the two oxide layers. Importantly, the radical

substituent in our atomistic model interacts with the aluminum oxide layer, which is expected to be the most relevant interaction for molecules embedded in an aluminum oxide layer as well.

II. The HOMO and LUMO peaks in the d*I*/d*V* plots are, by design, measured under substantial external bias. This will influence the electronic structure of the molecules in the junction and the interfaces. The calculations, by contrast, are done for molecules in the absence of such an external bias/electric field. These differences can lead to deviations between the PDOS for our idealized atomistic model and the experimentally obtained d*I*/d*V* curves.

10. Effect of external magnetic field on the *I*-*V* and d*I*/d*V* curves of reference double-tunnel junction without any molecules

Figure S11a and **S11**b show the *I*-*V* and d*I*/d*V* curves of a reference Si-based double-tunnel junction without containing any molecules under magnetic field ranging from 0 T to 7 T. The device structure of the reference double-tunnel junction is shown in the inset of **Figure S11**a. No peak was observed in the d*I*/d*V* curves which indicate no molecules are present in the reference sample. No significant changes in tunneling current under magnetic field was observed. **Figure S11**c and **S11**d show MR curves of the reference sample. No MR was observed in reference sample.

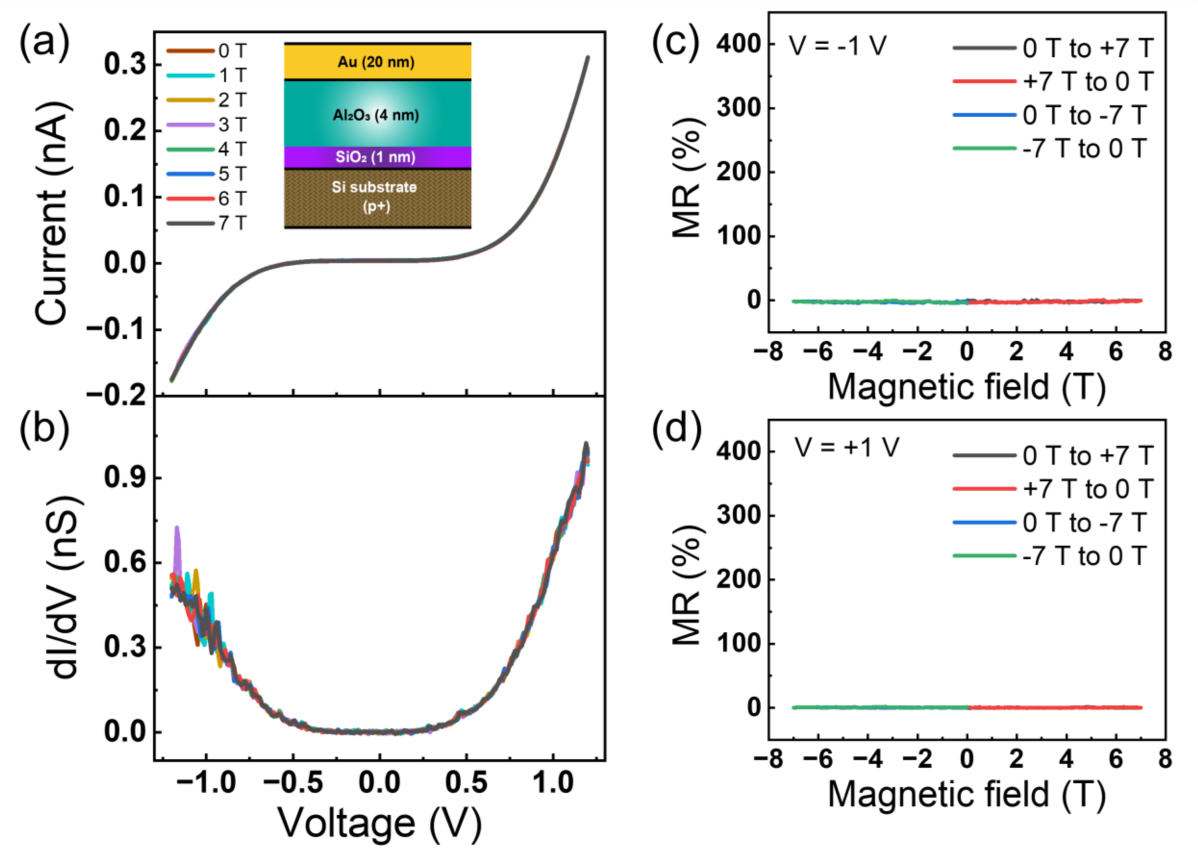

Figure S11. (a) *I-V* and (b) d*I*/d*V* curves of a reference Si-based double-tunnel junction without containing any molecules under magnetic field ranging from 0 T to 7 T. MR curves were measured at (c) −1 V and (d) 1 V in the reference sample. For (a)–(d) measurement temperature was fixed at 3 K.

11. Raw and 10-point smoothed data of *I-V* and d*I*/d*V* of TEMPO-OPE sample

To eliminate large noises in *I*–*V* and d*I*/d*V* curves, we implemented 10-point-data smoothing with Savitzky-Golay smoothing method in the raw data of *I*–*V* characteristics and corresponding d*I*/d*V* curves of TEMPO-OPE samples under magnetic field ranging from 0 T to 7 T at a temperature of 3 K. The comparison of *I*–*V* and d*I*/d*V* curves with the raw data and 10-ponit smoothed data are shown in **Figure S12**a-h.

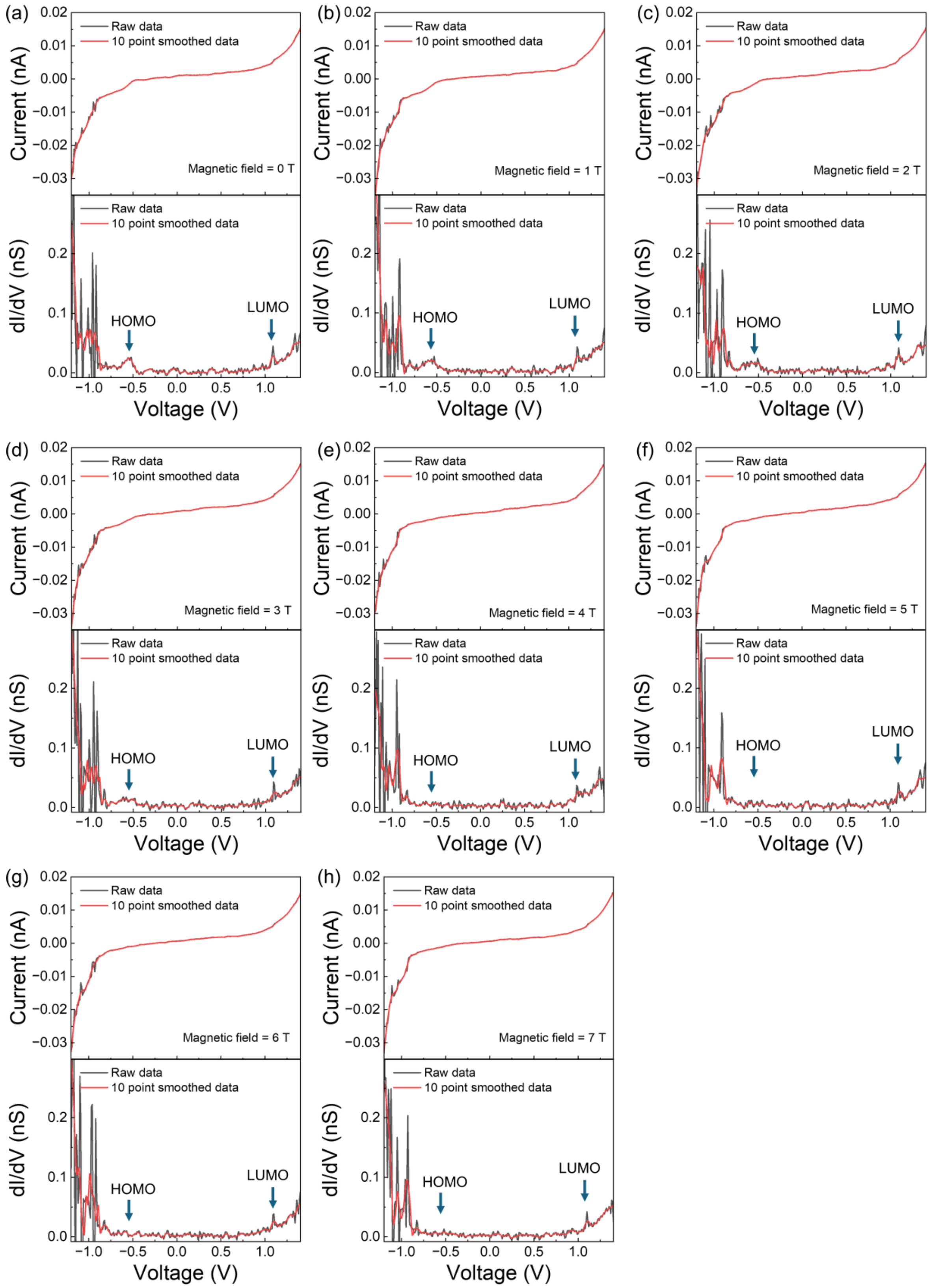
(a)
Raw data
10 point smoothed data
Magnetic field = 0 T
Current (nA)
dI/dV (nS)
HOMO
LUMO
Voltage (V)
(b)
Magnetic field = 1 T
(c)
Magnetic field = 2 T
(d)
Magnetic field = 3 T
(e)
Magnetic field = 4 T
(f)
Magnetic field = 5 T
(g)
Magnetic field = 6 T
(h)
Magnetic field = 7 T

Figure S12. Raw data and 10-point smoothed data of *I*–*V* characteristics and corresponding d*I*/d*V* curves of TEMPO-OPE sample under magnetic field ranging from 0 T to 7 T (a to h) at a temperature of 3 K.

12. *I*-*V* and d*I*/d*V* of TEMPO-OPE sample with magnetic fields applied

Figure S13a and **S13**b show the *I*–*V* and d*I*/d*V* curves of TEMPO-OPE sample under return sweep of magnetic field from 7 to 0 T. The same phenomenon observed in the forward sweep from 0 to 7 T, namely the reduction in tunneling current via the HOMO, appeared under the return sweep from 7 to 0 T.

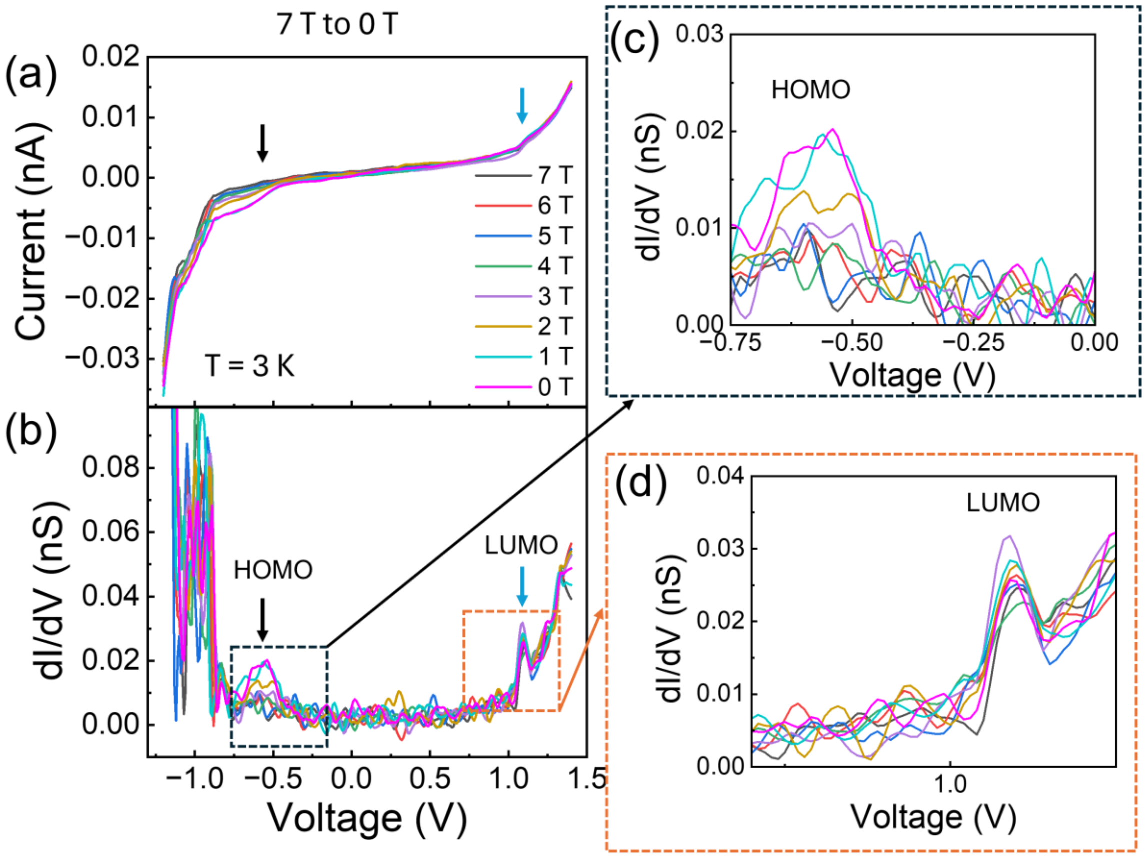

Figure S13. (a) *I*–*V* characteristics and corresponding (b) d*I*/d*V* curves of TEMPO-OPE sample under magnetic field ranging from 7 T to 0 T. The d*I*/d*V* peaks corresponding to the HOMO and LUMO levels of TEMPO-OPE molecules are shown in enlarged views with (c) black and (d) orange dotted rectangles, respectively.

To clarify the reduction in the HOMO d*I*/d*V* peak, the voltage positions of the d*I*/d*V* curves are plotted as a function of magnetic field in **Figure S14a** (forward magnetic-field sweep i.e., 0 T to 7 T) and **S14b** (reverse magnetic-field sweep i.e., 7 T to 0 T), where the d*I*/d*V* curves were numerical deviations of the *I*-*V* curves measured in the same device employed in Figure 2 in the manuscript and Figure S13. The d*I*/d*V* peak disappeared in magnetic fields of above 3 T.

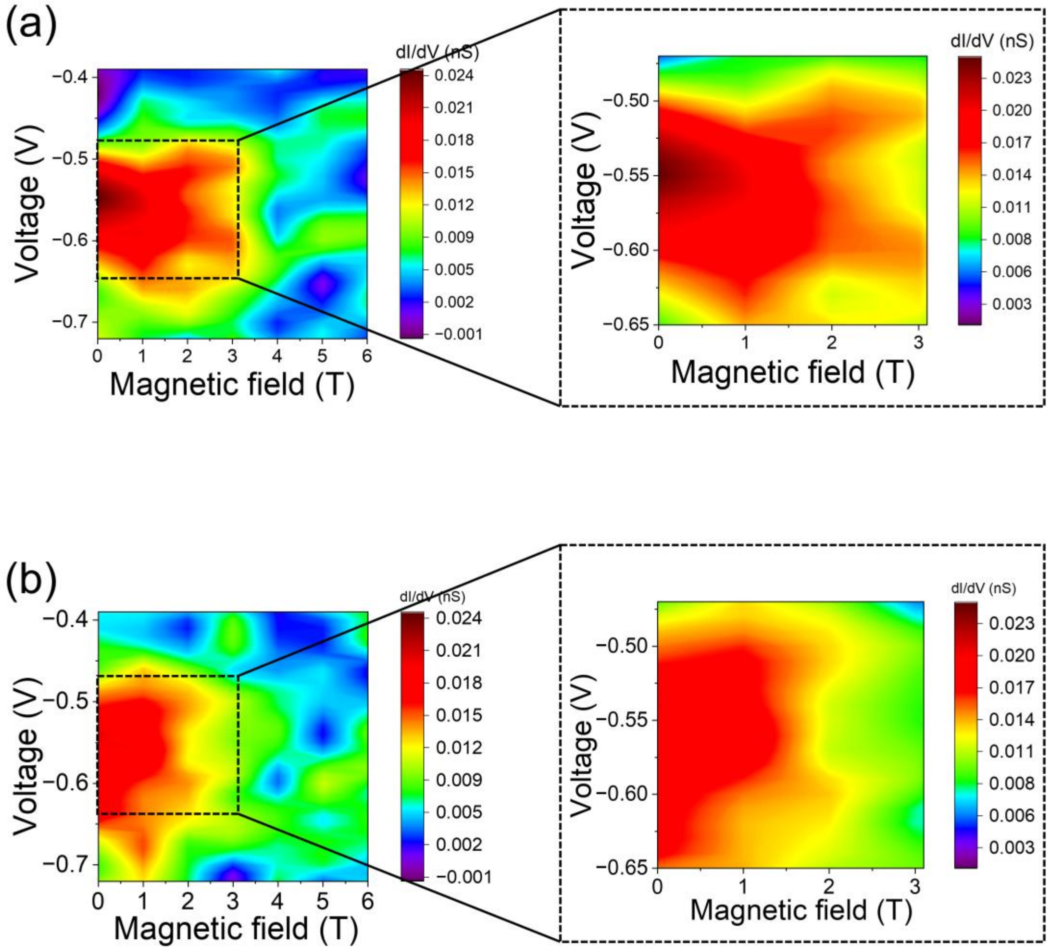

Figure S14. 2D color map of the d*I*/d*V* curves of (a) Figure 2c (forward magnetic-field sweep i.e., 0 T to 7 T) and (b) Figure S13c (reverse magnetic-field sweep i.e., 7 T to 0 T) plotted as a function of magnetic field and voltage.

13. Another example of *I-V* and d*I*/d*V* measurements in TEMPO-OPE sample under magnetic fields

Figure S15 shows the I–V and dI/dV curves measured under magnetic fields in the different device from that shown in Figure 2 in the manuscript. Similar reduction in only HOMO dI/dV peak was observed in the device. Conversely, no changes in the dI/dV peaks associated with HOMO−1 and LUMO were visible under magnetic fields up to 7 T.

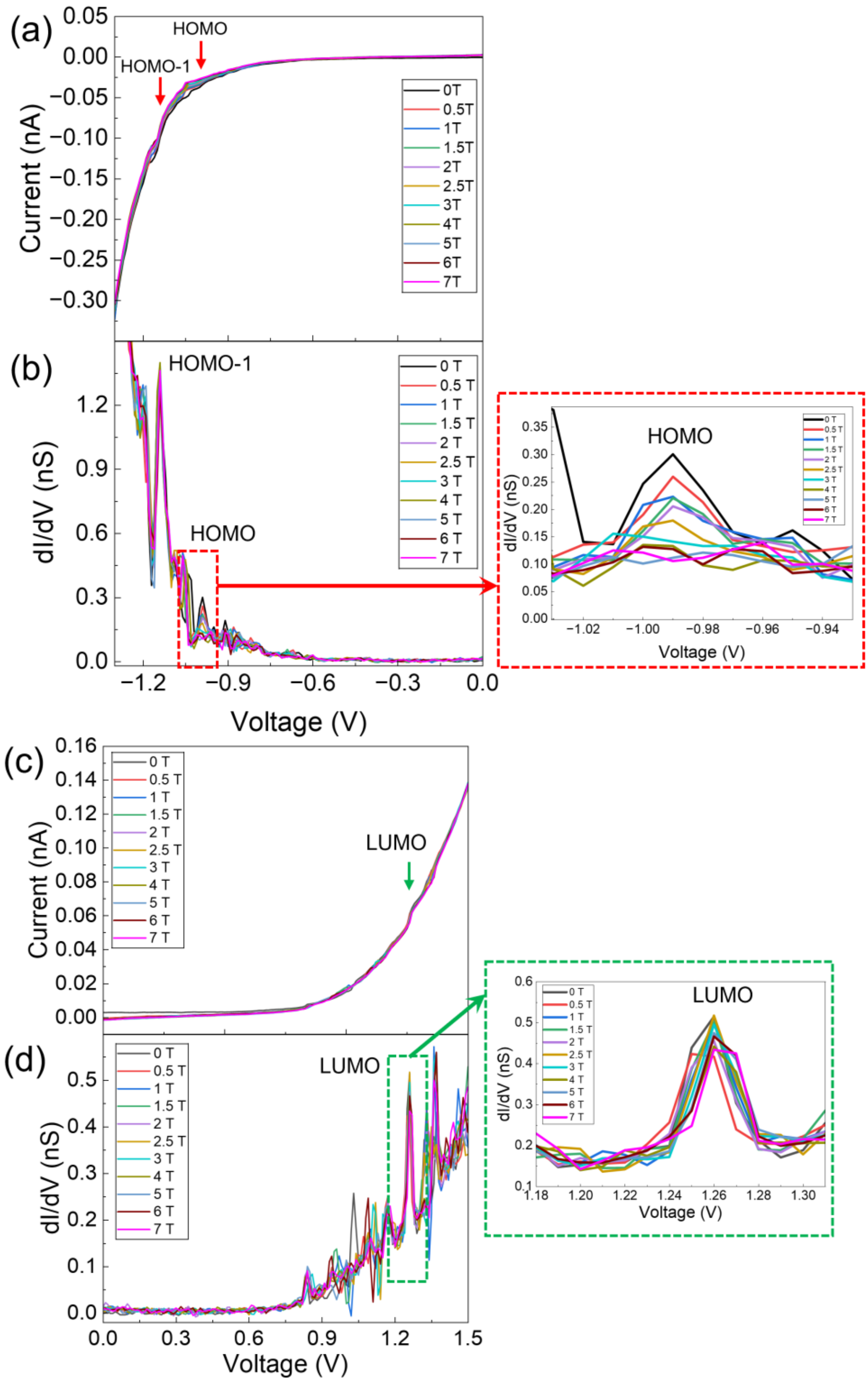

Figure S15. (a) *I-V* and (b) d*I*/d*V* curves of another TEMPO-OPE sample under magnetic field in the negative voltage region. The d*I*/d*V* peak corresponding to the HOMO was significantly suppressed under magnetic field; however, no splitting was observed. (c) *I-V* and (d) d*I*/d*V* curves of the same sample under magnetic field in the positive voltage region. No changes in the d*I*/d*V* peak corresponding to the LUMO was observed under magnetic field.

REFERENCES

(1) Hayakawa, R.; Karimi, M. A.; Wolf, J.; Huhn, T.; Zöllner, M. S.; Herrmann, C.; Scheer, E. Large Magnetoresistance in Single-Radical Molecular Junctions. *Nano Lett.* **2016**, *16* (8), 4960–4967.

(2) Hayakawa, R.; Hiroshiba, N.; Chikyow, T.; Wakayama, Y. Single-Electron Tunneling through Molecular Quantum Dots in a Metal-Insulator-Semiconductor Structure. *Adv. Funct. Mater.* **2011**, *21* (15), 2933–2937.

(3) Bera, J.; Kabdulov, M.; Wakayama, Y.; Huhn, T.; Hayakawa, R. Multilevel Resonant Tunneling through Purely Organic Radical Molecules in a Si-Based Double-Tunnel Junction. *ACS Appl. Mater. Interfaces* **2025**, *17* (15), 23018–23024.

(4) Kohn, W.; Sham, L. J. Self-Consistent Equations Including Exchange and Correlation Effects. *Phys. Rev.* **1965**, 140 (4A), A1133–A1138.

(5) Tirado-Rives, J.; Jorgensen, W. L. Performance of B3LYP Density Functional Methods for a Large Set of Organic Molecules. *J. Chem. Theory Comput.* **2008**, *4* (2), 297–306.

(6) Grimme, S.; Ehrlich, S.; Goerigk, L. Effect of the Damping Function in Dispersion Corrected Density Functional Theory. *J. Comput. Chem.* **2011**, *32* (7), 1456–1465.

(7) Zagorac, D.; Müller, H.; Ruehl, S.; Zagorac, J.; Rehme, S. Recent Developments in the Inorganic Crystal Structure Database: Theoretical Crystal Structure Data and Related Features. *J. Appl. Cryst.* **2019**, *52* (5), 918–925.

(8) Perdew, J. P.; Burke, K.; Ernzerhof, M. Generalized Gradient Approximation Made Simple. *Phys. Rev. Lett.* **1996**, *77* (18), 3865–3868.
